# B-DP: Dynamic Collection and Publishing of Continuous Check-In Data with Best-Effort Differential Privacy

**DOI:** 10.3390/e24030404

**Published:** 2022-03-14

**Authors:** Youqin Chen, Zhengquan Xu, Jianzhang Chen, Shan Jia

**Affiliations:** 1 State Key Laboratory of Information Engineering in Surveying, Mapping and Remote Sensing, Wuhan University, Wuhan 430079, China; chenyouqin@whu.edu.cn (Y.C.); jias@whu.edu.cn (S.J.); 2 College of Computer and Information Sciences, Fujian Agriculture and Forestry University, Fuzhou 350002, China; jzchen@fafu.edu.cn; 3 School of Computer Science and Engineering, University of Electronic Science and Technology of China, Chengdu 611731, China

**Keywords:** B-DP, DP, check-in data, dynamic collection and publishing

## Abstract

Differential privacy (DP) has become a de facto standard to achieve data privacy. However, the utility of DP solutions with the premise of privacy priority is often unacceptable in real-world applications. In this paper, we propose the best-effort differential privacy (B-DP) to promise the preference for utility first and design two new metrics including the point belief degree and the regional average belief degree to evaluate its privacy from a new perspective of preference for privacy. Therein, the preference for privacy and utility is referred to as expected privacy protection (EPP) and expected data utility (EDU), respectively. We also investigate how to realize B-DP with an existing DP mechanism (KRR) and a newly constructed mechanism (EXP${}_{Q}$) in the dynamic check-in data collection and publishing. Extensive experiments on two real-world check-in datasets verify the effectiveness of the concept of B-DP. Our newly constructed EXP${}_{Q}$ can also satisfy a better B-DP than KRR to provide a good trade-off between privacy and utility.

## 1. Introduction

The explosive progress of mobile Internet and location technology, LBS (Location Based Service) applications, including Brightkite, Gowalla, Facebook and other social network platforms, generate a large number of check-in data every day. Check-in data generally include information such as time, locations, PoI (Points of Interest) attributes, mood and comments, and hence the check-in data has become a carrier of a user’s life trajectory and interest tendency [1,2,3,4]. However, a data analyst’s mining and analysis of the check-in data may directly or indirectly expose the sensitive information of a data provider [5,6,7,8,9]. There have been many privacy protection methods [10,11,12,13,14,15,16]. Some of them [10,11] rely on specific attack assumptions and background knowledge, and some methods [12,13,14,15] are based on differential privacy (DP) [17]. DP provides provable privacy protection, which is independent of the background knowledge and computational power of an attacker. The protection level of DP is evaluated by privacy budget [17]. When the privacy budget is relatively small, it has strong privacy protection, but the utility is often poor [17]. With the gradual integration of DP on practical applications, utility has become the bottleneck of its development and popularization.

In general, there is a contradiction between privacy and utility and it is necessary to be a trade-off [18,19]. In [19], the authors discussed a monotone trade-off in the semi-honest model. Therein, when the utility becomes worse, the privacy protection becomes stronger, and on the other hand, when the utility gets better, the privacy protection gets weaker. In many other DP theoretical studies, including strict ϵ-DP [17] and relaxed (ϵ,δ)-DP [20], they often provide privacy priority and then make data more available or the best available, which is a kind of trade-off with satisfying utility as much as possible under the privacy guarantee. Unfortunately, the applications of DP in real-world do not seem to follow this principle completely. One of the best examples is the four applications in Apple’s MacOS Sierra (version 10.12), i.e., Emojis, New words, Deeplinks and Lookup Hints. When they collect the data, the privacy budget is set to only 1 or 2 per each datum, but the overall privacy budget for the four applications is as high as 16 per day [21]. Furthermore, Apple renews the available privacy budget every day, which would result in a potential privacy loss of 16 times the number of days that a user participated in DP data collection for the four applications [21]. It is far beyond the reasonable protection scope of DP [22].

Based on the above facts, when there exists a contradiction between privacy and utility, privacy is no longer a priority as suggested in the DP theoretical studies, but the most desirable way is to balance the preference for privacy and utility, where the preference for privacy and utility is referred to as expected privacy protection (EPP) and expected data utility (EDU), respectively. However, few researchers have proposed solutions to reasonably balance EPP and EDU except the authors in [23]. They proposed an adaptive DP and its mechanisms in a rational model, which can achieve a balance between the approximate EDU and the EPP by adding conditional filtering noise [23]. If the privacy protection intensity under the balance of the approximate EDU that is satisfied by the data analyst is not the expectation of the data provider, then it still cannot meet the EPP of the data provider. In addition, the absolute value range of the conditional filtering noise belongs to (0.5,1.5), which makes it easy to be attacked by background knowledge. Therefore, best-effort differential privacy (B-DP) is proposed to make the EDU satisfied first and then the EPP satisfied as much as possible in this paper. We face the following two basic challenges at least.

1. If the EDU is to be satisfied first, then privacy protection may be no longer to be guaranteed by DP, how does it evaluate the guarantee degree of satisfying EPP as much as possible under B-DP?
2. If there is a reasonable metric for the guarantee degree of satisfying EPP as much as possible under B-DP, does it exist an implementation mechanism (or algorithm) to realize B-DP?

With the challenges of B-DP above, this paper explores a typical application with dynamic collection and publishing of continuous check-in data, where the check-in scenario is a semi-honest model with an honest but curious data collector. Each check-in user visiting a POI generates a check-in state and perturbs his check-in state to a POI Center for his privacy protection, where the POI Center is a data collector. The frequency of check-in users are calculated by POI Center according to the received check-in states, which is approximately the check-in data distribution and used for publishing to data analysts. We assume that one check-in state is perturbed to only one check-in state and each publishing is required to satisfy the EDU first and then satisfy EPP as much as possible in the dynamic publishing, and moreover, the privacy to be protected is the check-in state of a user and the utility to be realized is the distribution of the check-in data with relative error as its metrics (see Section 4.1 for more details). In fact, since the relative error is used as a metric of the published distribution, it needs a distribution dependent privacy protection mechanism (or implementation) in order to satisfy the EPP as much as possible under the constraint of EDU. In addition, since each publishing is required to satisfy the EDU first and then satisfy EPP as much as possible in the dynamic publishing, it needs a algorithm to make the privacy protection under the constraint of EDU to be satisfied continuously as much as possible in the process of dynamic publishing. Therefore, this mechanism or algorithm will be proposed from a new perspective, which is different from the existing methods in literature.

### 1.1. Our Contributions

The main contributions of this paper are concluded as follows.

1. A privacy protection concept of B-DP and two metrics of privacy guarantee degree are put forward. B-DP discussed in this paper is an expansion of the concept of DP, which can satisfy the EDU first and then provide the EPP as much as possible to be usefull for real-world applications. It uses two new metrics including the point belief degree (see Definition 4) and the regional average belief degree (see Definition 5) to quantify the degree of privacy protection for any expected privacy budget (see Section 4.2), rather than for DP itself by the privacy budget ϵ to evaluate only one EPP with the expected privacy budget equal to ϵ. In addition, the regional average belief degree can be used as the average guarantee degree of the EPP in a region including multiple expected privacy budgets. To the best of our knowledge, it is a new discussion and definition of B-DP that is different from the existing literature, and it uses two new metrics to explore and analyze the performance of privacy from a new perspective of the preference for privacy.
2. An EXP${}_{Q}$ mechanism is proposed (see Definition 10). The newly constructed EXP${}_{Q}$ mechanism can be used to the categorical data for privacy protection, which smartly alters the privacy budget based on its probability in the data distribution to make itself to realize a better B-DP compared to the existing KRR mechanism [24,25]. Thereby, it also verifies that B-DP can be better realized to provide a good trade-off between privacy and utility.
3. The dynamic algorithm with the implementation algorithms of two perturbation mechanisms is proposed to realize the dynamic collection and publishing of continuous check-in data and meanwhile to satisfy B-DP. The two perturbation mechanisms include the newly constructed EXP${}_{Q}$ and a classical DP mechanism KRR [25,26] (a simple local differential privacy (LDP) mechanism). We take KRR as an example to show how to realize B-DP based on the existing DP mechanisms for the categorical data. Moreover, the number of domain values of both KRR and EXP${}_{Q}$ is more than 2 and both the randomized algorithms based on them only take one value as input and one value as output. In addition, the dynamic algorithm can also be used to other applications of social behavior except check-in data.

### 1.2. Outline

The remainder of this paper is organized as follows: Section 2 summarizes the related work on the trade-off methods, utility metrics of relative error and LDP mechanisms. Section 3 presents conceptual background of DP and details of KRR mechanism and utility metrics. Section 4 introduces the system model, the relevant definitions of B-DP, including two metrics of the guarantee degree, etc., and model symbolization of the check-in data. Section 5 introduces the design and implementation of B-DP mechanisms and Section 6 describes the design of B-DP mechanism algorithm in the dynamic collection and publishing. Section 7 provides the experimental evaluation of the dynamic collection and publishing algorithm based on both two B-DP mechanisms. Finally, we provide a discussion and conclusion in Section 8.

## 2. Related Work

DP has become a research hotspot in the field of privacy protection since Dwork [12] proposed it in 2006. The model of DP starts from the traditional centralization [15,18], gradually grows to be distributed [27], and develops to be localization [24,28] and even to be personalized localization [29] and so on. It is not only the evolution process of DP technique, but also the comprehensive embodiment of the gradual integration of DP technique with real-world applications. However, no matter how it evolves, the two themes running through DP are privacy and utility [18], which is also focused by this paper. Table 1 summarizes the mainly related work from the pespective of privacy and utility priority as well as their metrics, the used privacy mechanism and the focusing problem with EPP and EDU. It will be divided into three categories to show its details.

1. Trade-off model with utility first. The majority of DP research is based on the trade-off model with privacy first, while there are few relevant ones on the trade-off model with utility first. Therein, Katrina et al. [30] proposed a generalized “noise reduction” framework based on the modified “Above Threshold” algorithm [33] to minimize the empirical risk of privacy (ERM) on the premise of utility priority, but the scheme is only applicable to the framework that minimizes the empirical risk of privacy, where the privacy minimized may not be able to meet the EPP. Liu et al. proposed firstly that DP satisfies the monotonic trade-off between privacy and utility and its associated bounded monotone trade-off under the semi-honest model. They showed that there is no trade-off under the rational model, while unilateral trade-off could lead to utility disaster or privacy disaster [18,23,34]. They also presented an adaptive DP and its mechanisms under the rational model, which can realize the trade-off between approximately EDU and EPP by adding conditional filtering noise [23], but the mechanisms are probably not able to meet the expectation of data provider for privacy protection and are easily attacked by background knowledge because of the adding conditional filtering noise. Most importantly, the above two utility-first research [23,30] do not provide a quantitative metrics of the unmet privacy protection or the unmet degree of EPP, whereas this paper presents two detailed quantitative metrics including the point belief degree and the regional average belief degree to evaluate the privacy from a new perspective of preference for privacy.
2. Utility metrics of relative error. Maryam et al. [31] presented DP in real-world applications, which discussed how to add Laplace [12] noise from a view of utility. They studied the relationship between the cumulative probability of noise and the privacy level in Laplace mechanism and combined with the relative error metrics to discuss how to use a DP mechanism reasonably without losing the established utility. However, the literature does not delve into the details that how the guarantee degree of privacy protection will be changed when utility is satisfied. Xiao et al. [18] presented a DP publishing algorithm on a batch query using resampling technique of correlation noise to reduce noise added and improve data utility. When the algorithm picks the priority items each time, it is based on the intermediate results with noise, and the intermediate results with noise are not enough to reflect the original order of data. In this way, there is a bias in adjusting the privacy budget allocation, which may cause the query items that should be optimized to be not optimized, thus affecting the utility of published data. However, the literature is a classical example of optimizing utility with privacy first, which runs counter to the theme of this paper. In addition, the above two schemes are essentially based on the central DP and use continuous Laplace mechanism, which are different from the LDP (discrete) data statistics and release required by the check-in application in this paper. Therefore, these schemes cannot be directly applied to the applications this paper considers.
3. LDP mechanisms. In 1965, Warner first proposed the randomized response technique (W-RR) to collect statistical data on sensitive topics and keep the sensitive data of contributing individuals confidential [35]. Although W-RR can strictly satisfy ϵ-LDP [25] in one survey statistics, multiple collections on the same survey individuals will weaken the privacy protection intensity [12]. Therefore, Erlingsson et al. [28] used a double perturbation scheme combining permanent randomized response with instantaneous randomized response, namely, RAPPOR, to expand the application of W-RR, and it has been used by Google in Chrome browser to collect users’ behavior data. In addition, RAPPOR also uses Bloom Filter technology [36] as the encoding method, which maps the statistical attributes into a binary vector. Finally, the mapping relation and Lasso regression method [37] are combined to reconstruct the frequency statistics corresponding to the original attribute string. Due to the high communication cost of RAPPOR, Bassily et al. [32] proposed the S-Hist method. In the method, each user first encodes his attributes, then randomly selects one of the bits and uses the randomized response technique to perturb it, and finally sends the result of the perturbation to the data collector, so as to reduce the communication cost. Chen et al. [29] proposed a PCEP mechanism and designed a PLDP (personalized LDP) applied to spatial data with it, aiming to protect the users’ location information and count the number of users in the area. Therein, the privacy budget of the scheme is determined by the users’ personalization, and hence the utility depends on the users’ individual behavior settings. In addition, the mechanism combines the S-Hist [32] method and adopts the random projection technique [38]. Although it can greatly reduce the communication cost, it still has the problem of unstable query precision. Based on the check-in application with multiple check-in spots in this paper, the KRR mechanism [24,25] just easily fits this application with no prior data distribution knowledge, but it is not very good for B-DP. In addition, DP has already been studied in these applications, such as social networks [39,40], recommender systems [41], data publishing [42,43,44], deep learning [45], reinforcement learning [46] and federated learning [47].

## 3. Preliminaries

In this section, the key notations used in this paper are given in Table 2.

### 3.1. Differential Privacy (DP)

Differential privacy (DP), broadly speaking, is a privacy protection technique that does not depend on an attacker’s background knowledge and computational power [17,20,48]. It can be generally divided into central DP and LDP depending on whether it is based on a trusted data collector [33]. The formal definitions of these two types of DP are given as follows.

**Definition** **1** ((ϵ,δ)-(Central) DP [17,20])**.** *A randomized algorithm M and a set S of all possible outputs of M, for a given dataset D and any adjacent dataset $D^{\prime }$ that differ on at most one record, if M satisfies the following inequality, then it is said that M satisfies (ϵ,δ)-(central) DP.*

(1) $$P\left[M\left(D\right)\in S\right]\leq e^{\epsilon }\times P\left[M\left(D^{\prime }\right)\in S\right]+\delta ,$$

*where P[·] represents the risk of privacy disclosure and is controlled by the randomness of algorithm M, the parameter ϵ is called privacy budget that represents the level of privacy protection, and δ represents the probability of failure to satisfy ϵ-(central) DP. When δ=0, M satisfies the ϵ-(central) DP.*

**Definition** **2** ((ϵ,δ)-LDP [25,26])**.** *A randomized algorithm K, for a given dataset χ, any $x,x^{\prime }\in \chi$ and any y∈Range(K), is said to satisfy (ϵ,δ)-LDP if K satisfies*

(2) $$P\left[K\left(x\right)=y\right]\leq e^{\epsilon }\times P\left[K\left(x^{\prime }\right)=y\right]+\delta ,$$

*where P[·],ϵ and δ have the similar meanings as above in Definition 1.*

In the check-in application of this paper, the POI Center is an honest and curious data collector, even if the POI Center or other attackers can obtain the check-in state submitted by a user, they cannot conclusively infer the original check-in state of the user. If K can satisfy (ϵ,δ)-LDP to protect the check-in state of the user, then it needs to meet the following definition.

**Definition** **3** (Check-in state of (ϵ,δ)-LDP)**.** *A user u generates a check-in in a POI, whose check-in state variable is denoted as $s^{u}$ with $s^{u}\in \left\{S_{1},S_{2},\cdots ,S_{n}\right\}$. Assume that the original check-in state of u is $S_{j}$ or $S_{j^{\prime }}$ for $j,j^{\prime }\in \left[1,n\right]$. Moreover, $S_{j}$ and $S_{j^{\prime }}$ generate the same check-in state $S_{i}$ for i∈[1,n] after being perturbed by a randomized algorithm K, respectively, and the perturbed check-in state variable is ${\tilde{s}}^{u}$ with ${\tilde{s}}^{u}\in \left\{S_{1},S_{2},\cdots ,S_{n}\right\}$. If there exists an $\epsilon \in R^{+}$ such that K satisfies the following constraints for $i,j,j^{\prime }\in \left[1,n\right]$,*

(3) $$P\left({\tilde{s}}^{u}=S_{i}|s^{u}=S_{j}\right)\leq e^{\epsilon }P\left({\tilde{s}}^{u}=S_{i}|s^{u}=S_{j^{\prime }}\right)+\delta ,$$

*where, $P({\tilde{s}}^{u}=S_{i}|s^{u}=S_{j})$ and $P({\tilde{s}}^{u}=S_{i}|s^{u}=S_{j^{\prime }})$ are the perturbation probabilities of the original check-in states $S_{j}$ and $S_{j^{\prime }}$ to the check-in state $S_{i}$, respectively, then K will enable the check-in state to satisfy (ϵ,δ)-LDP. When δ=0, K satisfies ϵ-LDP.*

**Property** **1** (Parallel composition [49])**.** *Assume that randomized algorithms are $K_{1},K_{2},\cdots ,K_{n}$ and their privacy budgets are $\epsilon_{1},\epsilon_{2},\cdots ,\epsilon_{n}$, respectively, then for the disjoint datasets $D_{1},D_{2},\cdots ,$ $D_{n}$, an algorithm $K(K_{1}\left(D_{1}\right),K_{2}\left(D_{2}\right),\cdots ,K_{n}\left(D_{n}\right))$ provides max$\left(\epsilon_{i}\right)$-(local) DP, and the level of privacy protection it provides depends on the largest privacy budget.*

### 3.2. KRR Mechanism

KRR is a LDP mechanism [24,25], which satisfies the following probability distribution,

(4) $$P\left(y|x\right)=\frac{1}{e^{\epsilon }+k-1}\left\{\begin{array}{c} e^{\epsilon },y=x\\ 1,y\neq x \end{array}\right.$$

where x,y∈χ and |χ|=k.

KRR is a more general form of the randomized response mechanism of W-RR, that is, when k=2, KRR degenerates into W-RR.

### 3.3. Utility Metrics

In this paper, the worst relative error of POIs in check-in statistics will be used to measure the overall utility of the check-in application, where the calculation formula of the relative error of $POI_{i}$ is as follows,

(5) $$err\left(r_{i},r_{i}^{*}\right)=\frac{|r_{i}-r_{i}^{*}|}{max\{r_{i},\phi \}},$$

where $r_{i}^{*}$ is the estimated result of $POI_{i}$ check-in statistics after LDP protection, $r_{i}$ is the real check-in result of the $POI_{i}$, and the parameter ϕ is a constant to avoid the situations that $r_{i}=0$ causes the denominator to be 0 or $r_{i}$ is too small [18,50,51]. For the convenience of analysis, this paper will use the relative root mean square error for the utility metrics approximately, the specific formula is as follows,

(6) $$err\left(r_{i},r_{i}^{*}\right)=\frac{\sqrt{\xi (r_{i},r_{i}^{*})}}{max\{r_{i},\phi \}},$$

where $\xi (r_{i},r_{i}^{*})$ is the expectation of the mean square error between the real statistical result $r_{i}$ and the statistical estimate result $r_{i}^{*}$ after LDP protection, and the parameter ϕ is defined as above. Then, the formula for calculating the maximum relative error of *n* POIs is as follows,

(7) $$err\left(r,r^{*}\right)=max\left(err\left(r_{i},r_{i}^{*}\right)\right)=max\left(\frac{\sqrt{\xi (r_{i},r_{i}^{*})}}{max\{r_{i},\phi \}}\right).$$

As above, $r_{i},r_{i}^{*}$ not only can represent data distribution, but also can represent frequency or counts.

## 4. Problem Formulations

### 4.1. System Model

**Figure 1:**
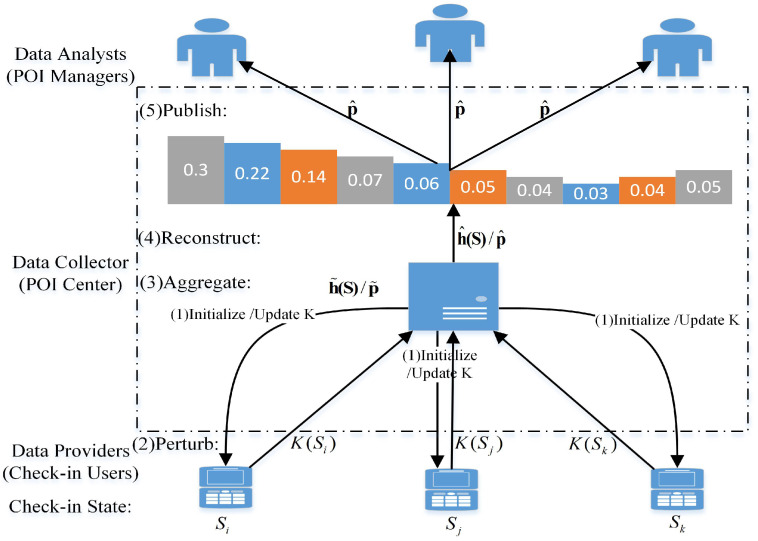
POI check-in model. Therein, $S_{i},S_{j}$ and $S_{k}$ represent check-in states. $\tilde{h}\left(S\right)$ and $\tilde{p}$ represent the check-in counts and the check-in frequency (data distribution) in perturbation phase, respectively, while $\hat{h}\left(S\right)$ and $\hat{p}$ represent the check-in counts and the check-in frequency (data distribution) in construction phase, respectively. *K* represents a perturbation mechanism. The more details can also be seen in Section 4.3.

As shown in Figure 1, there are three types of participants, namely, check-in users (data providers), POI Center (data collector), and data analysts (for example, POI managers) in the check-in model. Each check-in user visiting a POI generates a check-in state and sends it to POI Center through a terminal with the check-in APP, where each check-in state corresponds to a count and the check-in state belongs to catagory data. POI Center calculates the counts and frequency of check-in users’ visiting POIs according to the received check-in states, where frequency is approximately the check-in data distribution and used for publishing to data analysts. In addition, it is assumed that each check-in user is independent of each other and only one check-in state is submitted by a check-in user in one publishing. It is also assumed that the check-in scenario is a semi-honest model, in which the POI Center is an honest but curious data collector, and the check-in state of a user is sensitive. Hence, a user will adopt a perturbation mechanism (for example, LDP mechanism) to perturb his check-in state for his privacy protection, and then sends it to the POI Center. Therein, it is assumed that one check-in state is perturbed to only one check-in state.

In this paper, we focus on the dynamic collection and publishing of continuous check-in data with both privacy and utility requirements, where the privacy to be protected is the check-in state of a user and the utility to be realized is the distribution of the check-in data with relative error as its metrics. Therein, the privacy refers to EPP, which is the preference for privacy of a user, and the utility refers to EDU, which is the preference for utility of a data analyst. Moreover, each publishing is required to satisfy the EDU first and then satisfy EPP as much as possible in the dynamic publishing. Thereby, we adopt B-DP based on the LDP model including perturbation, aggregation, reconstruction and publishing, and we also need to have the process of initializing or updating the perturbation mechanism *K* at least to make every publishing to satisfy the EPP as much as possible under the EDU satisfied first in the dynamic publishing, as shown in Figure 1.

### 4.2. The Related Concepts of B-DP

In the concept of best-effort differential privacy (B-DP), there is an expected privacy protection (EPP) and an expected data utility (EDU), respectively. When the two cannot be satisfied simultaneously, the EDU should be satisfied first and the EPP should be satisfied as much as possible. Since the protection level of DP is evaluated by privacy budget [17], the preference for privacy also refers to the preference for the privacy budget in the B-DP. Hence, the EPP refers to a data provider’s preference for the privacy budget and we define this privacy budget as the expected privacy budget symbolized as $\epsilon_{e}$. We use $Region(\epsilon_{e})$ to symbolize the expected privacy protection region, which refers to a data provider’s preference for a region including multiple expected privacy budgets.

We use η to symbolize the EDU. In this paper, the expectation of the maximum relative error of Formula (7) is used to measure data utility. When the expectation of the maximum relative error of Formula (7) is less than or equal to η, it means that the EDU is satisfied; when equal, it means that the EDU is just satisfied. The privacy budget of a DP mechanism that just satisfies the EDU η is symbolized as $\epsilon_{\eta }$.

**Definition** **4** ($C_{\epsilon_{e}}$-Point belief degree)**.** *It defines the guarantee degree of EPP under the expected privacy budget $\epsilon_{e}$, which can be provided by the $\epsilon_{\eta }$-DP mechanism, as the point belief degree, and the symbol is denoted as $C_{\epsilon_{e}}$. Moreover, $C_{\epsilon_{e}}=\sum_{i=1}^{n}{\tilde{p}}_{i}\chi \left(\epsilon_{i},\epsilon_{e}\right)$, where n represents the number of POIs in check-in application, ${\tilde{p}}_{i}$ represents the probability of $POI_{i}$ perturbed by $\epsilon_{\eta }$-DP mechanism, $\epsilon_{i}$ represents the actual privacy budget of $POI_{i}$, and $\chi (\epsilon_{i},\epsilon_{e})$ represents an indicator function for whether the EPP is satisfied, which is defined as follows,*

(8) $$\chi \left(\epsilon_{i},\epsilon_{e}\right)=\left\{\begin{array}{c} 0,\epsilon_{i}>\epsilon_{e}\;\\ 1,\epsilon_{i}\leq \epsilon_{e} \end{array}\right..$$

**Definition** **5** ($C_{Region(\epsilon_{e})}$-Regional average belief degree)**.** *The average guarantee degree of the EPP under the expected privacy protection region $Region(\epsilon_{e})$, which can be provided by the $\epsilon_{\eta }$-DP mechanism, is defined as the regional average belief degree, and the symbol is denoted as $C_{Region(\epsilon_{e})}$. When $Region\left(\epsilon_{e}\right)=\left\{\epsilon_{e_{1}},\epsilon_{e_{2}},\cdots ,\epsilon_{e_{K}}\right\}$ and $\epsilon_{e_{1}}<\epsilon_{e_{2}}<\cdots <\epsilon_{e_{K}}$ for K≥2, it defines*

(9) $$C_{Region(\epsilon_{e})}=\frac{1}{\epsilon_{e_{K}}-\epsilon_{e_{1}}}\sum_{k=1}^{K-1}\left(\epsilon_{e_{k+1}}-\epsilon_{e_{k}}\right)C_{\epsilon_{e_{k}}},$$

*where $C_{\epsilon_{e_{k}}}$ can refer the definition of point belief degree.*

**Definition** **6** ($\left(\epsilon_{\eta },C_{\epsilon_{e}}\right)$-B-DP)**.** *The DP mechanism that just satisfies the EDU η with the point belief degree $C_{\epsilon_{e}}$ of the expected privacy budget $\epsilon_{e}$ is defined as $\left(\epsilon_{\eta },C_{\epsilon_{e}}\right)$-B-DP. Therein, the $\left(\epsilon_{\eta },C_{\epsilon_{e}}\right)$-B-DP, where the point belief degree $C_{\epsilon_{e}}$ is maximum, is defined as $\left(\epsilon_{\eta },C_{\epsilon_{e}}\right)$-Best-B-DP.*

**Definition** **7** ($\left(\epsilon_{\eta },C_{Region(\epsilon_{e})}\right)$-B-DP)**.** *The DP mechanism that just satisfies the EDU η with the regional average belief degree $C_{Region(\epsilon_{e})}$ of the expected privacy protection region $Region(\epsilon_{e})$ is defined as $\left(\epsilon_{\eta },C_{Region(\epsilon_{e})}\right)$-B-DP. Therein, the $(\epsilon_{\eta },$ $C_{Region(\epsilon_{e})})$-B-DP, where the regional average belief degree $C_{Region(\epsilon_{e})}$ is maximum, is defined as $\left(\epsilon_{\eta },C_{Region(\epsilon_{e})}\right)$-Best-B-DP.*

Note that, generally, B-DP includes both central B-DP and local B-DP, which depends on whether it is based on a trusted data collector the same as the DP. This paper focuses on local B-DP.

### 4.3. Model Symbolization

Let $POI_{i}$ with i∈[1,n] represent *n* POIs in check-in scenario, and the check-in state space is $S=\{S_{1},S_{2},\cdots ,S_{n}\}$ where $S_{i}$ is the check-in state of $POI_{i}$. Let $s^{u},{\tilde{s}}^{u},{\hat{s}}^{u}\in S$ be variables of the original check-in state, the perturbed check-in state and the estimated check-in state of the user *u*, respectively. Let $p,\tilde{p}$ and $\hat{p}$ be the probability distributions of the original check-ins, the perturbed check-ins and the estimated check-ins, respectively, where $p={\left[p_{1},p_{2},\cdots ,p_{n}\right]}^{T}$, $\tilde{p}={\left[{\tilde{p}}_{1},{\tilde{p}}_{2},\cdots ,{\tilde{p}}_{n}\right]}^{T}$ and $\hat{p}={\left[{\hat{p}}_{1},{\hat{p}}_{2},\cdots ,{\hat{p}}_{n}\right]}^{T}$. Assume that it is the same probability distribution law for all the users, that is, $p_{i}=P\left(s^{u}=S_{i}\right)$, ${\tilde{p}}_{i}=P\left({\tilde{s}}^{u}=S_{i}\right)$ and ${\hat{p}}_{i}=P\left({\hat{s}}^{u}=S_{i}\right)$ for any i∈[1,n] and *u*. $h\left(S\right)={\left[h\left(S_{1}\right),h\left(S_{2}\right),\cdots ,h\left(S_{n}\right)\right]}^{T}$, $\tilde{h}\left(S\right)={\left[\tilde{h}\left(S_{1}\right),\tilde{h}\left(S_{2}\right),\cdots ,\tilde{h}\left(S_{n}\right)\right]}^{T}$ and $\hat{h}\left(S\right)={\left[\hat{h}\left(S_{1}\right),\hat{h}\left(S_{2}\right),\cdots ,\hat{h}\left(S_{n}\right)\right]}^{T}$ represent the original check-in counts vector, the perturbed check-in counts vector and the estimated check-in counts vector with $m\in N^{+}$ users, respectively.

**Definition** **8** (Random perturbation and perturbation probability matrix *Q*)**.** *The process for any user u to change check-in state from $S_{j}$ to $S_{i}$ with a certain perturbation probability is called random perturbation, and the perturbation probability is denoted as $q_{ij}=P\left({\tilde{s}}^{u}=S_{i}|s^{u}=S_{j}\right)$ with $S_{i},S_{j}\in S$. The matrix composed of $q_{ij}$ for any i,j∈[1,n] is called the perturbation probability matrix Q, where $Q={\left(q_{ij}\right)}_{n\times n}$ and $\sum_{i=1}^{n}q_{ij}=1$ for any j∈[1,n].*

Therefore, the perturbed probability distribution $\tilde{p}$, the original probability distribution p and the perturbation probability matrix *Q* have the following relationship

(10) $$\begin{array}{cc} \tilde{p} & =Qp. \end{array}$$

From Equation (10), it can be seen that $\tilde{p_{i}}=\sum_{j=1}^{n}q_{ij}p_{j}$ for any i∈[1,n]. Obviously, $\tilde{p_{i}}$ and $p_{i}$ are not always equal, and hence the result of the perturbation is biased. Assume that *Q* is always reversible and its inverse matrix is defined as $R=Q^{-1}={\left(r_{ij}\right)}_{n\times n}$. Therefore, it can get the following theorem.

**Theorem** **1.** *The check-in counts vector h(S) is perturbed by the perturbation probability matrix Q to obtain the perturbed check-in counts vector $\tilde{h}\left(S\right)$ and then it is corrected by the inverse matrix R. The estimated check-in counts vector $\hat{h}\left(S\right)=R\tilde{h}\left(S\right)$ satisfies $E[\hat{h}\left(S\right)]=h\left(S\right)$.*

**Proof.**  Since $E\left[\hat{h}\left(S\right)\right]=E\left[R\tilde{h}\left(S\right)\right]=E\left[RQh\left(S\right)\right]$, and RQ=I, it has $E\left[\hat{h}\left(S\right)\right]=E\left[h\left(S\right)\right]=h\left(S\right)$. Therefore, the result follows. □

Theorem 1 states that the estimated check-in counts vector $\hat{h}\left(S\right)$ obtained after the correction of the inverse matrix *R* is an unbiased estimate of the original check-in counts vector h(S).

Here, the relative root mean square error $err(h\left(S_{i}\right),\hat{h}\left(S_{i}\right))$ of the original check-in counts $h(S_{i})$ and the estimated check-in counts $\hat{h}\left(S_{i}\right)$ for *n* POIs and *m* users on $POI_{i}$ can be calculated as follows,

(11) $$\begin{array}{c} err\left(h\left(S_{i}\right),\hat{h}\left(S_{i}\right)\right)=\frac{\sqrt{Var[\hat{h}\left(S_{i}\right)]}}{E[\hat{h}\left(S_{i}\right)]}, \end{array}$$

where ϕ=1 and $Var[\hat{h}\left(S_{i}\right)]$ can be calculated as follows,

(12) $$\begin{array}{c} Var\left[\hat{h}\left(S_{i}\right)\right]=\sum_{j=1}^{n}r_{ij}^{2}\left(\sum_{k=1}^{n}q_{jk}h\left(S_{k}\right)\right)-h\left(S_{i}\right). \end{array}$$

**Theorem** **2.** *The relative root mean square error between the original probability $p_{i}$ of $POI_{i}$ check-ins and the estimated probability ${\hat{p}}_{i}$ of $POI_{i}$ check-ins is $err\left(p_{i},{\hat{p}}_{i}\right)=\frac{\sqrt{Var[\hat{h}\left(S_{i}\right)]}}{E[\hat{h}\left(S_{i}\right)]}$, where i∈[1,n].*

**Proof.**  The relative root mean square error between the original probability $p_{i}$ of $POI_{i}$ check-ins and estimated probability ${\hat{p}}_{i}$ of $POI_{i}$ check-ins can be represented as follows,

(13) $$\begin{array}{c} err\left(p_{i},{\hat{p}}_{i}\right)=\frac{\sqrt{E[{\left(p_{i}-{\hat{p}}_{i}\right)}^{2}]}}{p_{i}}=err\left(h\left(S_{i}\right),\hat{h}\left(S_{i}\right)\right). \end{array}$$

Therefore, $err\left(p_{i},{\hat{p}}_{i}\right)=\frac{\sqrt{Var[\hat{h}\left(S_{i}\right)}]}{E[\hat{h}\left(S_{i}\right)]}$, where i∈[1,n]. □

According to Formulas (11)–(13), it can be known $err(p_{i},{\hat{p}}_{i})$, *Q* and p are related.

If $max(err\left(p_{i},{\hat{p}}_{i}\right))=\eta$ means that the EDU is just satisfied, then *Q* should satisfy the following constraints according to B-DP.

(14) $$\begin{array}{cc} \; & max\left(err\left(p_{i},{\hat{p}}_{i}\right)\right)\leq \eta ,i\in \left[1,n\right],\\ \; & q_{ij}\leq e^{\epsilon_{i}}q_{ij^{\prime }},\forall i,j,j^{\prime }\in \left[1,n\right],\epsilon_{i}\geq 0,\\ \; & \sum_{i=1}^{n}q_{ij}=1,\forall j\in \left[1,n\right]. \end{array}$$

Assume that there is a randomized algorithm K with a perturbation probability matrix *Q*, which can provide the expected privacy budget $\epsilon_{e}$ with the point belief degree $C_{\epsilon_{e}}=\sum_{i=1}^{n}{\tilde{p}}_{i}\chi \left(\epsilon_{i},\epsilon_{e}\right)$. Then, if K wants to satisfy $\left(\epsilon_{\eta }=max\left(\epsilon_{i}\right),C_{\epsilon_{e}}\right)$-Best-B-DP, it should still need to maximize $C_{\epsilon_{e}}$. Therefore, K should satisfy the following optimization problem.

(15) $$\begin{array}{cc} \; & \underset{Q}{maximize}C_{\epsilon_{e}}\\ \; & s.t.max\left(err\left(p_{i},{\hat{p}}_{i}\right)\right)\leq \eta ,i\in \left[1,n\right],\\ \; & q_{ij}\leq e^{\epsilon_{i}}q_{ij^{\prime }},\forall i,j,j^{\prime }\in \left[1,n\right],\epsilon_{i}\geq 0,\\ \; & \sum_{i=1}^{n}q_{ij}=1,\forall j\in \left[1,n\right]. \end{array}$$

Similarly, it is assumed that K can provide the expected privacy protection region $Region\left(\epsilon_{e}\right)=\left\{\epsilon_{e_{1}},\epsilon_{e_{2}},\cdots ,\epsilon_{e_{K}}\right\}$ with the regional average belief degree $C_{Region(\epsilon_{e})}=\frac{1}{\epsilon_{e_{K}}-\epsilon_{e_{1}}}\sum_{k=1}^{K-1}\left(\epsilon_{e_{k+1}}-\epsilon_{e_{k}}\right)C_{\epsilon_{e_{k}}}$, where $C_{\epsilon_{e_{k}}}$ is the point belief degree of the expected privacy budget $\epsilon_{e_{k}}$. Then, if K wants to satisfy $\left(\epsilon_{\eta }=max\left(\epsilon_{i}\right),C_{Region(\epsilon_{e})}\right)$-Best-B-DP, it should still need to maximize $C_{Region(\epsilon_{e})}$. Therefore, K should satisfy the following optimization problem.

(16) $$\begin{array}{cc} \; & \underset{Q}{maximize}C_{Region(\epsilon_{e})}\\ \; & s.t.max\left(err\left(p_{i},{\hat{p}}_{i}\right)\right)\leq \eta ,i\in \left[1,n\right],\\ \; & q_{ij}\leq e^{\epsilon_{i}}q_{ij^{\prime }},\forall i,j,j^{\prime }\in \left[1,n\right],\epsilon_{i}\geq 0,\\ \; & \sum_{i=1}^{n}q_{ij}=1,\forall j\in \left[1,n\right]. \end{array}$$

From the above optimization Equations (15) and (16), in each optimization problem, it can be concluded that the perturbation probability matrix *Q* contains $n^{2}$ unknown variables, $n^{3}$ inequality constraints, *n* equality constraints and one EDU η constraint. Therefore, directly solving the optimization problem is a huge challenge, especially in the case of a large domain size *n*. Therefore, two simplified models are considered in this paper and we will present the details one by one in the following section.

## 5. Design and Implementation of B-DP Mechanism

This section includes the design of two B-DP mechanisms and their implementation algorithms. One is based on a classical LDP mechanism KRR, and the other is based on the newly constructed mechanism EXP${}_{Q}$ in this paper. The number of domain values of both two mechanisms is more than 2. Moreover, we combine three data distributions with the typical non-uniformity and two B-DP mechanisms to directly show and analyze the two metrics proposed in this paper, including the point belief degree and the regional average belief degree.

### 5.1. B-DP Mechanism Based on KRR

Without prior knowledge of the data distribution, we assume that it is uniform. Setting $\epsilon_{i}=\epsilon_{j}=\epsilon_{\eta },$$q_{ii}=p,q_{ij}=q$ for i,j∈[1,n],j≠i and $q_{ii}\geq q_{ij}$, then it has $p=\frac{e^{\epsilon_{\eta }}}{e^{\epsilon_{\eta }}+n-1},q=\frac{1}{e^{\epsilon_{\eta }}+n-1}$, and $err(p,\hat{p})=\eta$. Therefore, the privacy budget of KRR here is not arbitrary, which is constrained by the EDU of $err(p,\hat{p})=\eta$.

**Definition** **9** ($\epsilon_{\eta }$-KRR)**.** *KRR that just meets the EDU η is called $\epsilon_{\eta }$-KRR.*

Here, it can also derive the following theorem.

**Theorem** **3.** *If there exists $\epsilon_{\eta }$-KRR, then it satisfies $\epsilon =\epsilon_{\eta }$-LDP. Moreover, the point belief degree of $\epsilon_{\eta }$-KRR is*

(17) $$C_{\epsilon_{e}}=\left\{\begin{array}{c} 0,\epsilon_{e}<\epsilon_{\eta }\\ 1,\epsilon_{e}\geq \epsilon_{\eta } \end{array}.\right.$$

**Proof.**  If there exists $\epsilon_{\eta }$-KRR, then it has $p=\frac{e^{\epsilon_{\eta }}}{e^{\epsilon_{\eta }}+n-1},q=\frac{1}{e^{\epsilon_{\eta }}+n-1}$. Since $\epsilon =ln\left(\frac{p}{q}\right)=\epsilon_{\eta }$, it satisfies $\epsilon =\epsilon_{\eta }$-LDP. According to $C_{\epsilon_{e}}=\sum_{i=1}^{n}{\tilde{p}}_{i}\chi \left(\epsilon_{i},\epsilon_{e}\right)$, when $\epsilon_{e}<\epsilon_{\eta }$, $\chi \left(\epsilon_{i},\epsilon_{e}\right)=\chi \left(\epsilon_{\eta },\epsilon_{e}\right)=0$, it has $C_{\epsilon_{e}}=0$; when $\epsilon_{e}\geq \epsilon_{\eta }$, $\chi \left(\epsilon_{i},\epsilon_{e}\right)=\chi \left(\epsilon_{\eta },\epsilon_{e}\right)=1$, it has $C_{\epsilon_{e}}=1$. Hence, the result follows. □

Thus, it is impossible for $\epsilon_{\eta }$-KRR to provide the EPP $\epsilon_{e}$, or provide the EPP $\epsilon_{e}$ with a 100% satisfaction. Therefore, what KRR can achieve is two distinct jumps of EPP with or without guarantee, that is, it is not a good B-DP mechanism.

### 5.2. B-DP Mechanism Based on EXP${}_{Q}$

Since relative error is used as utility metrics about the check-in data distribution in this paper, and a privacy budget of DP usually determines absolute error which is the numerator of relative error, thus the privacy budget of every POI should vary with its probability in the distribution of check-ins, that is, the value of the privacy budget should be reduced when the corresponding probability in the data distribution becomes larger, and increased when the corresponding probability in the data distribution becomes smaller. In this way, the small amounts of check-ins can also satisfy the EDU, while the large amounts of check-ins can also satisfy the EPP in priority, so as to better realize B-DP. It defines the following perturbation mechanism EXP${}_{Q}$.

**Definition** **10** (Perturbation mechanism EXP${}_{Q}$). *Given the data distribution $p=[p_{1},p_{2},\cdots ,$$p_{n}{]}^{T}$, where $p_{1}\geq p_{2}\geq \cdots \geq p_{n}$. Call the randomized algorithm with Q as perturbation mechanism EXP${}_{Q}$ if Q satisfies $q_{ij}\propto e^{-\gamma u(j,i)}$, where $q_{ij}$ is the probability that the check-in state is perturbed from $S_{j}$ to $S_{i}$ for i,j∈[1,n] and γ≥0 is the privacy setting parameter, where u(j,i) satisfies Formulas (18)–(20), and $\kappa_{n}\in \left[0,n\right]$ is the parameter of privacy protection intensity change point.*

*(i) When $\kappa_{n}=0$,*

(18) $$u\left(j,i\right)=\left\{\begin{array}{c} 0,i=j\\ 1+p_{n-i+1},i\neq j \end{array}.\right.$$

*(ii) When $\kappa_{n}\in \left[1,n-1\right]$,*

(19) $$u\left(j,i\right)=\left\{\begin{array}{c} 0,i=j\\ 1-p_{i},i\neq j\;\mathrm{and}\;i\leq \kappa_{n}\\ 1+p_{n-i+\kappa_{n}+1},i\neq j\;\mathrm{and}\;i>\kappa_{n} \end{array}.\right.$$

*(iii) When $\kappa_{n}=n$,*

(20) $$u\left(j,i\right)=\left\{\begin{array}{c} 0,i=j\\ 1-p_{i},i\neq j \end{array}.\right.$$

**Theorem** **4.** *In perturbation mechanism EXP${}_{Q}$, its Q satisfies the following properties.*

*(i) When $\kappa_{n}=0$ and the normalized factor perturbing from $POI_{j}$ to a POI is $\Omega_{j}=1+\sum_{k=1,k\neq j}^{n}e^{-\gamma (1+p_{n-k+1})}$ for i,j∈[1,n],*

(21) $$q_{ij}=\left\{\begin{array}{c} \frac{1}{\Omega_{j}},i=j\\ \frac{e^{-\gamma (1+p_{n-i+1})}}{\Omega_{j}},i\neq j \end{array}.\right.$$

(ii) When $\kappa_{n}\in \left[1,n-1\right]$ and the normalized factor perturbing from $POI_{j}$ to a POI is $\Omega_{j}=1+\sum_{k=1,k\neq j}^{\kappa_{n}}e^{-\gamma (1-p_{k})}+\sum_{k=\kappa_{n}+1,k\neq j}^{n}e^{-\gamma (1+p_{n-k+\kappa_{n}+1})}$ for i,j∈[1,n],

(22) $$q_{ij}=\left\{\begin{array}{c} \frac{1}{\Omega_{j}},i=j\\ \frac{e^{-\gamma (1-p_{i})}}{\Omega_{j}},i\neq j\;\mathrm{and}\;i\leq \kappa_{n}\\ \frac{e^{-\gamma (1+p_{n-i+\kappa_{n}+1})}}{\Omega_{j}},i\neq j\;\mathrm{and}\;i>\kappa_{n} \end{array}.\right.$$

(iii) When $\kappa_{n}=n$ and the normalized factor perturbing from $POI_{j}$ to a POI is $\Omega_{j}=1+\sum_{k=1,k\neq j}^{n}e^{-\gamma (1-p_{k})}$ for i,j∈[1,n],

(23) $$q_{ij}=\left\{\begin{array}{c} \frac{1}{\Omega_{j}},i=j\\ \frac{e^{-\gamma (1-p_{i})}}{\Omega_{j}},i\neq j \end{array}.\right.$$

**Proof.**  According to Definition 10, it can be seen that $q_{ij}\propto e^{-\gamma u(j,i)}$ is the probability that the check-in state of $POI_{j}$ is perturbed to that of $POI_{i}$ for i,j∈[1,n]. Moreover, since $\sum_{k=1}^{n}q_{kj}=1$, it is easy to obtain the result of Theorem 4. □

**Definition** **11.** *($\epsilon_{\eta }$-EXP${}_{Q}$) EXP${}_{Q}$ that just satisfies the EPU η is called $\epsilon_{\eta }$-EXP${}_{Q}$ where $\epsilon_{\eta }=max_{1\leq i\leq n}\left(\epsilon_{i}\right)$ and $\epsilon_{i}$ is the actual privacy budget for each $POI_{i}$, that is, $e^{-\epsilon_{i}}\leq \frac{q_{ij}}{q_{ij^{\prime }}}\leq e^{\epsilon_{i}}$ for $j,j^{\prime }\in \left[1,n\right]$.*

**Theorem** **5.** *Based on the definition of $\epsilon_{\eta }$-EXP${}_{Q}$,*

*(1) if there exists $\epsilon_{\eta }$-EXP${}_{Q}$, then it satisfies $\epsilon =\epsilon_{\eta }$-LDP;*

*(2) when $\kappa_{n}$ is fixed and the point belief degree of $\epsilon_{\eta }$-EXP${}_{Q}$ is $C_{\epsilon_{e}}=\sum_{i=1}^{n}{\tilde{p}}_{i}\chi \left(\epsilon_{i},\epsilon_{e}\right)$, where $\epsilon_{i}$ is the actual privacy budget for each $POI_{i}$, $\epsilon_{\eta }$-EXP${}_{Q}$ is the approximately optimal $\left(\epsilon_{\eta },C_{\epsilon_{e}}\right)$-Best-B-DP, where the indicator function $\chi (\epsilon_{i},\epsilon_{e})$ is*

(24) $$\chi \left(\epsilon_{i},\epsilon_{e}\right)=\left\{\begin{array}{c} 0,\epsilon_{i}>\epsilon_{e}\\ 1,\epsilon_{i}\leq \epsilon_{e} \end{array}\right.;$$

*(3) if there exists $\epsilon_{\eta }$-EXP${}_{Q}$ and its point belief degree is $C_{\epsilon_{e}}$, then it satisfies $\left(\epsilon_{e},1-C_{\epsilon_{e}}\right)$-LDP.*

**Proof.**  See Appendix A. □

### 5.3. Implementation of B-DP Machanism

For the check-ins scenario, two B-DP mechanisms based on KRR and EXP${}_{Q}$ are proposed and realized in this paper. KRR is one of the classical mechanisms of LDP, but it cannot well realize B-DP. EXP${}_{Q}$ is newly proposed in this paper, which can not only provide the protection of approximately optimal $\left(\epsilon_{\eta },C_{\epsilon_{e}}\right)$-Best-B-DP, but also provides the protection of relaxed $\left(\epsilon_{e},1-C_{\epsilon_{e}}\right)$-LDP to satisfy the EDU. The pseudo codes of the two B-DP mechanisms are given in Algorithms 1 and 2, respectively.

**Algorithm 1** B-DP machanism based on KRR.**Input:** Probability distribution $p={\left[p_{1},p_{2},\cdots ,p_{n}\right]}^{T}$, sample size *m* and expected data utility (EDU) η**Output:** Privacy budget $\epsilon_{\eta }$ and perturbation probability matrix *Q*
 1:Initialize $\epsilon_{\eta }>0$, iteration step size $\Delta \epsilon_{\eta }>0$ and the worst utility MaxRE=1; 2:
**while** MaxRE>η **do**
 3: *Q* is constructed by KRR with $\epsilon_{\eta }$; 4: According to the relative error formula, the current worst relative error CurrentMaxRE $=max_{i\in [1,n]}\left(\frac{\sqrt{\sum_{j=1}^{n}r_{ij}^{2}\left(\sum_{k=1}^{n}q_{jk}p_{k}\right)-mp_{i}}}{mp_{i}}\right)$ is obtained, where $R={\left(r_{i^{\prime }j^{\prime }}\right)}_{n\times n}=Q^{-1},$$Q={\left(q_{i^{\prime }j^{\prime }}\right)}_{n\times n}$, referred to Formulas (11)–(12) and Theorems 1 and 2 for details. 5: **if** CurrentMaxRE<MaxRE **then** 6:  MaxRE=CurrentMaxRE; 7: **end if** 8: **if** MaxRE>η **then** 9:  $\epsilon_{\eta }=\epsilon_{\eta }+\Delta \epsilon_{\eta }$;10: **end if**11:
**end while**
12:**return**$\epsilon_{\eta }$ and *Q*


**Algorithm 2** B-DP mechanism based on EXP${}_{Q}$.**Input:** Probability distribution $p={\left[p_{1},p_{2},\cdots ,p_{n}\right]}^{T}$, sample size *m*, expected data utility (EDU) η, expected privacy budget $\epsilon_{e}$ (or expected privacy protection region $Region\left(\epsilon_{e}\right)=\left\{\epsilon_{e_{1}},\epsilon_{e_{2}},\cdots ,\epsilon_{e_{K}}\right\}$ with $\epsilon_{e_{1}}<\epsilon_{e_{2}}<\cdots <\epsilon_{e_{K}}$)**Output:** Privacy setting parameter $\gamma_{\eta }$, the parameter of privacy protection intensity change point $\kappa_{n}$, perturbation probability matrix *Q* and actual privacy budget $\epsilon_{i}$ of $POI_{i}$ for i∈[1,n]
 1:Initialize privacy setting parameter $\gamma_{0}>0$ and the iteration step size $\Delta \gamma_{\eta }>0$; 2:Initialize $\kappa_{n}=n$ and tag=0, where tag is used to identify whether it exists a comparatively reasonable result or not; 3:
**while** $\kappa_{n}\geq 0$ **do**
 4: Initialize $\gamma_{\eta }=\gamma_{0}$, $\epsilon_{i}=0$ for i∈[1,n], the worst utility MaxRE=1 and $C_{\epsilon_{e}}=0$ (here, the initialization of regional average belief degree $C_{Region(\epsilon_{e})}$ is also uniformly recorded as $C_{\epsilon_{e}}=0$); 5: **while** MaxRE>η **do** 6:  *Q* is constructed by EXP${}_{Q}$ with $p,\gamma =\gamma_{\eta }$ and $\kappa_{n}$, where the row represents the perturbed check-in state and the column represents the original check-in state; 7:  According to *Q*, use $ln\frac{max(q_{ij})}{min(q_{ij^{\prime }})}$ to update the value $\epsilon_{i}$, where $Q={\left(q_{ij}\right)}_{n\times n}$ and $i,j,j^{\prime }\in \left[1,n\right]$; 8:  According to the relative error formula, the current worst relative error is obtained $CurrentMaxRE=max_{i\in [1,n]}$$\left(\frac{\sqrt{\sum_{j=1}^{n}r_{ij}^{2}\left(\sum_{k=1}^{n}q_{jk}p_{k}\right)-mp_{i}}}{mp_{i}}\right)$, where $R={\left(r_{i^{\prime }j^{\prime }}\right)}_{n\times n}=Q^{-1}$, referred to Formulas (11)–(12) and Theorems 1 and 2 for details; 9:  **if** CurrentMaxRE<MaxRE **then**10:   MaxRE=CurrentMaxRE;11:  **end if**12:  **if** MaxRE>η **then**13:   $\gamma_{\eta }=\gamma_{\eta }+\Delta \gamma_{\eta }$;14:  **end if**15: **end while**16: **if** $\epsilon_{i}$ is not all zero for i∈[1,n] **then**17:  Calculate the current point belief degree value according to $C_{\epsilon_{e}}^{*}=\sum_{i=1}^{n}{\tilde{p}}_{i}\chi \left(\epsilon_{i},\epsilon_{e}\right)$$=\sum_{i=1}^{n}\sum_{j=1}^{n}$$q_{ij}p_{j}\chi \left(\epsilon_{i},\epsilon_{e}\right)$, and set $C_{\epsilon_{e}}^{♯}=C_{\epsilon_{e}}^{*}$ (or, calculate the current regional average belief degree value according to $C_{Region(\epsilon_{e})}^{*}=\frac{1}{\epsilon_{e_{K}}-\epsilon_{e_{1}}}\sum_{k=1}^{K-1}$$\left(\epsilon_{e_{k+1}}-\epsilon_{e_{k}}\right)C_{\epsilon_{e_{k}}}$, where $C_{\epsilon_{e_{k}}}=\sum_{i=1}^{n}{\tilde{p}}_{i}\chi \left(\epsilon_{i},\epsilon_{e_{k}}\right)=\sum_{i=1}^{n}\sum_{j=1}^{n}q_{ij}p_{j}\chi \left(\epsilon_{i},\epsilon_{e_{k}}\right)$, and set $C_{\epsilon_{e}}^{♯}=C_{Region(\epsilon_{e})}^{*}$);18:  **if** $C_{\epsilon_{e}}<C_{\epsilon_{e}}^{♯}$ **then**19:   Update tag=1, $C_{\epsilon_{e}}=C_{\epsilon_{e}}^{♯}$, $\gamma_{opt}=\gamma_{\eta }$, $\kappa_{opt}=\kappa_{n}$, $Q_{opt}=Q$ and $\epsilon_{i}^{opt}=\epsilon_{i}\left(i\in \left[1,n\right]\right)$;20:  **end if**21: **end if**22: Update $\kappa_{n}=\kappa_{n}-1$;23:
**end while**
24:
**if** tag=0 **then**
25: Update $\kappa_{n}=0$, and update the value $\epsilon_{i}$ according to Step 7;26:
**else**
27: Record $\gamma_{\eta }=\gamma_{opt}$, $\kappa_{n}=\kappa_{opt}$, $Q=Q_{opt}$ and $\epsilon_{i}=\epsilon_{i}^{opt}\left(i\in \left[1,n\right]\right)$;28:
**end if**
29:**return**$\gamma_{\eta }$, $\kappa_{n}$, *Q* and $\epsilon_{i}$ for i∈[1,n]


### 5.4. Case Analysis of Point Belief Degree and Regional Average Beleif Degree

The above description theoretically analyzes two metrics, including the point belief degree and the regional average belief degree, on the two B-DP mechanisms based on KRR and EXP${}_{Q}$. In order to show the two metrics more clearly, the following of this section will use three data distributions with typical non-uniformity for analysis. For simplicity, in the following of this section, the KRR-based B-DP mechanism is represented by KRR and the EXP${}_{Q}$-based B-DP mechanism is represented by EXP${}_{Q}$, including the diagram descriptions.

(1) Three data distributions with typical non-uniformity.

**Figure 2:**
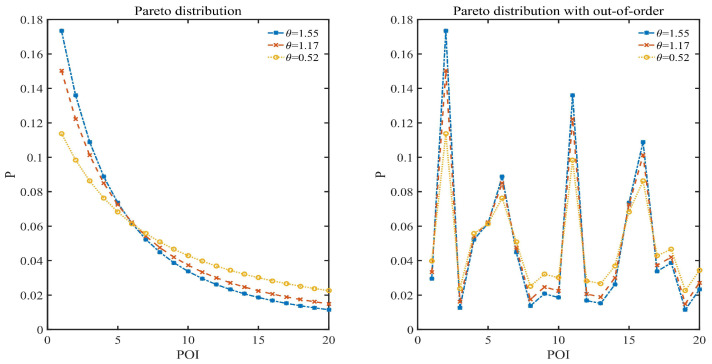
Pareto distribution.

The data distribution in this section is set as Pareto distribution, where the discrete case of Pareto distribution satisfies $p_{j}\propto \frac{1}{x_{j}^{\theta +1}}$ for j∈[1,n],θ>0 and $x_{j}>0$. Three data distributions of n=20, θ= 1.55, 1.17 and 0.52 are shown in Figure 2 and are, respectively, denoted as P1, P2 and P3, where $x_{j}=x_{1}+\left(j-1\right)\Delta x,x_{1}=2,\Delta x=0.2$. Figure 2 shows both ordered and disordered cases of Pareto distribution, where the disordered case illustrates that the identification of scenic spots is independent of the order of probability. It also shows the corresponding Gini coefficient of P1, P2 and P3, which is calculated according to the method of Gini mean difference [52]. Gini coefficient is used to indicate the degree of unevenness of data distribution. There exists a quantitative relationship between Pareto distribution parameter and Gini coefficient in Table 3. As shown in Figure 2, the data distribution of θ=1.55 is pretty uneven, and the data distribution of θ=1.17 is relatively reasonably uneven, while the data distribution of θ=0.52 is relatively even.

(2) Point belief degree

**Figure 3:**
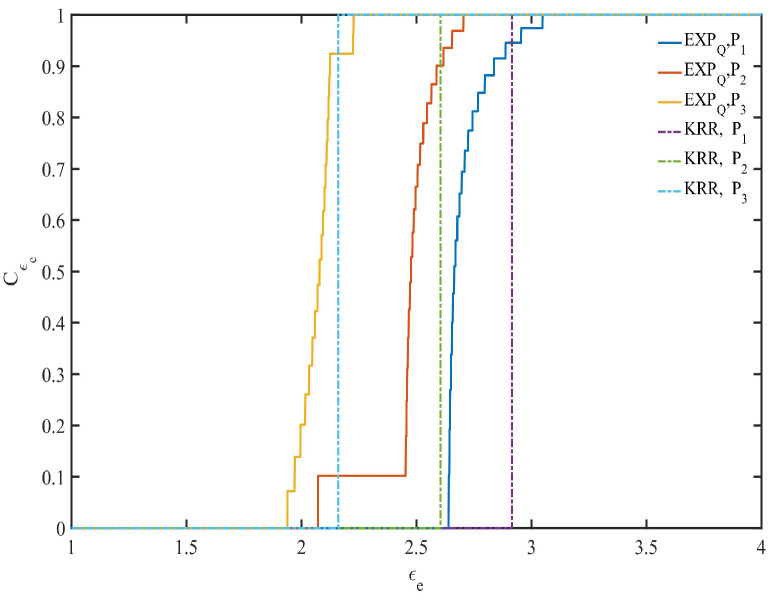
Point belief degree $\left(C_{\epsilon_{e}}\right)$ with η=0.1.

In the point belief degree $C_{\epsilon_{e}}$ in KRR, let $\epsilon_{e}=\epsilon_{\eta }$ ($\epsilon_{\eta }$ is used as the expected privacy budget or used as a basis for division of the expected privacy protection region, just for better comparison between KRR and EXP${}_{Q}$) determined by $\epsilon_{\eta }$-KRR (see definition of $\epsilon_{\eta }$-KRR and Algorithm 1 for details), which equals the $\epsilon_{e}$-coordinate of the jump point shown by the dotted line in Figure 3. It is also combined with the same $\epsilon_{e}$ and η to determine the perturbation probability and related parameters with EXP${}_{Q}$ (see Algorithm 2 for details). For example, when the EDU η=0.1, the point belief degree $C_{\epsilon_{e}}$ of KRR and EXP${}_{Q}$ is shown in Figure 3.

From Figure 3, it can be seen that under the same EDU, if the expected privacy budget of any data provider is $\epsilon_{e}\geq \epsilon_{\eta }$, KRR can provide $\epsilon_{e}$-DP with belief degree of 1. On the other hand, if the expected privacy budget of any data provider is $\epsilon_{e}<\epsilon_{\eta }$, its belief degree is 0. However, in EXP${}_{Q}$, if the expected privacy budget of any data provider is $\epsilon_{e}\geq \epsilon_{\eta }$, it indicates that it cannot satisfy the EPP when $\epsilon_{e}$ is closer to $\epsilon_{\eta }$, and when $\epsilon_{e}$ is large enough, it can also provide $\epsilon_{e}$-DP with belief degree of 1. Conversely, if the expected privacy budget of all data providers is $\epsilon_{e}<\epsilon_{\eta }$, it indicates that it can satisfy the EPP when $\epsilon_{e}$ is closer to $\epsilon_{\eta }$, and the degree of providing $\epsilon_{e}$-DP is greater when $\epsilon_{e}$ is closer to $\epsilon_{\eta }$. Therefore, in the case of EDU first, EXP${}_{Q}$ can provide a privacy guarantee degree between 0 and 1 for the EPP, while KRR can only provide either 0 or 1. Moreover, EXP${}_{Q}$ can provide more privacy protection than KRR, especially when the EDU and the EPP are contradictory, and when the EPP of all data providers is not fully (partially) satisfied.

(3) Regional average belief degree

In the regional average belief degree $C_{Region(\epsilon_{e})}$ in KRR, maximizing $C_{Region(\epsilon_{e})}$ equals to maximize $C_{\epsilon_{e}}$, and hence it is the same as Algorithm 1. According to the approximately optimal expected privacy budget $\epsilon_{\eta }$ under satisfying the EDU η, the data provider’s expected privacy protection region $Region(\epsilon_{e})$ can be roughly divided into three categories: $\left\{\forall \epsilon_{e}\in Region\left(\epsilon_{e}\right)>\epsilon_{\eta }\right\}$, $\left\{\forall \epsilon_{e}\in Region\left(\epsilon_{e}\right)<\epsilon_{\eta }\right\}$, and $\left\{\left\{\epsilon_{\eta }\right\}\subset Region\left(\epsilon_{e}\right)\right\}$.

**Figure 4:**
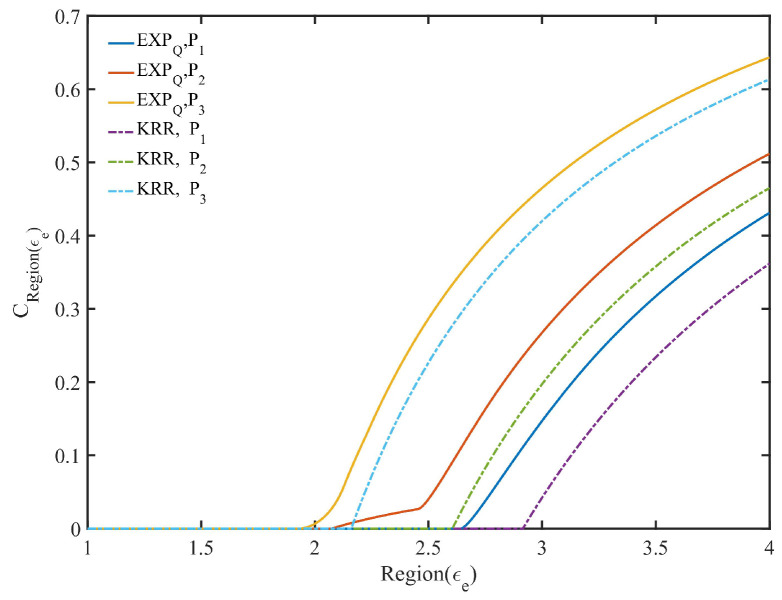
Regional average belief degree$\left(C_{Region(\epsilon_{e})}\right)$ with η=0.1.

Similarly, EXP${}_{Q}$ can provide different levels of optimal privacy protection for the three categories of expected privacy protection region (see Algorithm 2 for details). Generally speaking, the regional average privacy protection degree of EXP${}_{Q}$ in region $\left\{\forall \epsilon_{e}\in Region\left(\epsilon_{e}\right)>\epsilon_{\eta }\right\}$ is less than or equal to that of KRR. However, the regional average privacy protection degree of EXP${}_{Q}$ in region $\left\{\forall \epsilon_{e}\in Region\left(\epsilon_{e}\right)<\epsilon_{\eta }\right\}$ is greater than or equal to that of KRR. For region $\left\{\left\{\epsilon_{\eta }\right\}\subset Region\left(\epsilon_{e}\right)\right\}$, it may exist the situation where there is a contradiction between the EPP and the EDU. As shown in Figure 4, there is the regional average belief degree $C_{Region(\epsilon_{e})}$ of both mechanisms with $Region\left(\epsilon_{e}\right)=\left[1,1.001,1.002,\cdots ,4\right]$ and the data distributions P1, P2 and P3, respectively, where $\epsilon_{\eta }$ is determined by $\epsilon_{\eta }$-KRR with η=0.1 (see the dotted line in Figure 4 where the value of $\epsilon_{e}$ whose $C_{Region(\epsilon_{e})}$ is the first non-zero value is equal to $\epsilon_{\eta }$).

As can be seen from Figure 4, under the same EDU and the same expected privacy protection region, EXP${}_{Q}$ is more capable of offering data providers with a certain degree of privacy protection than KRR.

## 6. B-DP Dynamic Collection and Publishing Algorithm Design

Algorithms 1 and 2 are implemented with KRR and EXP${}_{Q}$ under the known data distribution, and moreover, the point belief degree and the regional average belief degree under B-DP are analyzed. In real-world, there is often no prior data distribution at the beginning or accurate prior data distribution cannot be obtained. This means the implementation of two B-DP mechanisms of Algorithms 1 and 2 cannot be directly applied to the collection and publishing of continuous check-in data with relative error as utility metrics. Therefore, this paper designs an iterative update algorithm to adaptively update the data distribution in order to realize the two B-DP mechanisms, so as to adaptively realize B-DP dynamic collection and publishing of continuous check-in data. See pseudo codes of Algorithm 3 for more details. Therein, Algorithms 1 or 2 is a main part of Algorithm 3.

**Algorithm 3** B-DP dynamic collection and publishing of check-in data algorithm—(KRR/EXP${}_{Q}$).
**Initialization process**: The data collector initializes the perturbation probability matrix *Q*, and the estimated data distribution ${\hat{p}}^{\left(0\right)}={\left[{\hat{p}}_{1}^{\left(0\right)},{\hat{p}}_{2}^{\left(0\right)},\cdots ,{\hat{p}}_{n}^{\left(0\right)}\right]}^{T}$, and the perturbation probability matrix *Q* is broadcasted to the data provider.1: For KRR, initialize $p_{i}=\frac{1}{n}$ for i∈[1,n] and $\epsilon_{\eta }=ln\left(\frac{1+\left(n-1\right)\sqrt{\frac{n-1}{m\eta^{2}+n-1}}}{1-\sqrt{\frac{n-1}{m\eta^{2}+n-1}}}\right)$; For EXP${}_{Q}$, initialization $p_{i}=\frac{1}{n}$ for i∈[1,n], $\kappa_{n}=0$ and $\gamma =\frac{n}{n+1}ln\left(\frac{1+\left(n-1\right)\sqrt{\frac{n-1}{m\eta^{2}+n-1}}}{1-\sqrt{\frac{n-1}{m\eta^{2}+n-1}}}\right)$;2: *Q* is constructed according to KRR/EXP${}_{Q}$;3: Initialize ${\hat{p}}_{i}^{\left(0\right)}=0$ for i∈[1,n];4: *Q* is broadcasted to the data provider.
**Perturbation process**
1: The data provider uses *Q* to perturb the check-in data;2: The perturbed check-in data are sent to the data collector.
**Statistics and update processes**

**(1) Statistical process: including aggregation and reconstruction procedures**
1: After collecting the check-in data of time slice *T*, the data collector carries out frequency statistics to get the perturbed data distribution $\tilde{p}$. Assuming that the current time slice is the *t*th, it is recorded as ${\tilde{p}}_{t}$;2: From the inverse estimation formula ${\hat{p}}_{t}=Q^{-1}{\tilde{p}}_{t}$, it obtains the estimated distribution ${\hat{p}}_{t}$;3: Correcting the estimated data distribution ${\hat{p}}_{t}$, it gets the corrected estimate data distribution ${\hat{p}}^{\left(t\right)}$ of the *t*th time slice (to be released);
**if** ${\hat{p}}^{\left(t-1\right)}\neq 0$ **then**
 ${\hat{p}}^{\left(t\right)}=\left(1-w\right){\hat{p}}^{\left(t-1\right)}+w{\hat{p}}^{\left(t\right)}$, where w∈(0,1) is the corrected estimate parameter of a positive real number;
**else**
 ${\hat{p}}^{\left(t\right)}={\hat{p}}_{t}$;
**end if**

**(2) Update process**
1: The data collector calculates maximum relative error $Re=max(|\frac{{\hat{p}}^{\left(t\right)}-{\hat{p}}^{\left(t-1\right)}}{{\hat{p}}^{\left(t-1\right)}}|)$ based on ${\hat{p}}^{\left(t\right)}$ and ${\hat{p}}^{\left(t-1\right)}$;2: Initialize $Re_{thredthold}\in \left(0,1\right)$, which is a update threshold parameter of a positive real number;
**if** $Re>Re_{thredthold}$ **then**
 2-1: Start a process of updating *Q* to get a new *Q*, as shown in Algorithm 1/Algorithm 2, where the input data distribution of Algorithm 1/Algorithm 2 is ${\hat{p}}^{\left(t\right)}$; 2-2: The data collector broadcasts the new *Q* to the data provider.
**end if**



Since the original data distribution is assumed to be uniform during initialization, it is possible to calculate the privacy setting parameter $\epsilon_{\eta }$ or γ with a closed-form expression that satisfies the EDU η, as shown in the example with EXP${}_{Q}$ below. According to Corollary A1 of Appendix A, in the case of uniform data distribution, EXP${}_{Q}$ degenerates into KRR. Let $\kappa_{n}=0$, the probability $q_{ij}$ of *Q* be calculated as follows, where $\gamma_{\eta }$ is γ that makes $max(err\left(p_{i},{\hat{p}}_{i}\right))=\eta$ true.

(25) $$q_{ij}=\left\{\begin{array}{c} \frac{e^{\gamma_{\eta }\left(1+\frac{1}{n}\right)}}{e^{\gamma_{\eta }\left(1+\frac{1}{n}\right)}+n-1},i=j\\ \frac{1}{e^{\gamma_{\eta }\left(1+\frac{1}{n}\right)}+n-1},i\neq j \end{array}.\right.$$

Let $p=\frac{e^{\gamma_{\eta }\left(1+\frac{1}{n}\right)}}{e^{\gamma_{\eta }\left(1+\frac{1}{n}\right)}+n-1},q=\frac{1}{e^{\gamma_{\eta }\left(1+\frac{1}{n}\right)}+n-1}$, and the inverse matrix *R* of *Q* can be expressed as

(26) $$r_{ij}=\left\{\begin{array}{c} \frac{1-q}{p-q},i=j\\ -\frac{q}{p-q},i\neq j \end{array}.\right.$$

Therefore, *p*, *q* and $\gamma_{\eta }$ that satisfy the EDU η can be calculated. Since p≥q, it has $q=-\frac{1}{n}\sqrt{\frac{n-1}{m\eta^{2}+n-1}}+\frac{1}{n}$ and $p=\frac{n-1}{n}\sqrt{\frac{n-1}{m\eta^{2}+n-1}}+\frac{1}{n}$. Hence, $\gamma_{\eta }=\frac{n}{n+1}ln\left(\frac{p}{q}\right)$, and $\gamma =\gamma_{\eta }=\frac{n}{n+1}ln\left(\frac{1+\left(n-1\right)\sqrt{\frac{n-1}{m\eta^{2}+n-1}}}{1-\sqrt{\frac{n-1}{m\eta^{2}+n-1}}}\right)$.

## 7. Experimental Evaluation of B-DP Dynamic Collection and Publishing Algorithm

In this paper, the check-in data uses relative error as its utility metrics and the implementation of the two B-DP mechanisms based on KRR and EXP${}_{Q}$ needs to rely on the data distribution. Therein, the number of domain values of both KRR and EXP${}_{Q}$ is more than 2, and moverover, both the randomized algorithms based on them only take one value as input and one value as output. Thereby, KRR and EXP${}_{Q}$ are fit for the check-in perturbation model we consider in this paper. In this section, we evaluate the performance of the dynamic algorithm based on the two B-DP mechanisms in terms of validity and robustness as well as privacy and utility. For simplicity, in the following of this section, we use KRR and EXP${}_{Q}$ to represent B-DP mechanism based on KRR and B-DP mechanism based on EXP${}_{Q}$ in the dynamic algorithm, respectively, including the diagram descriptions.

### 7.1. Experimental Settings

(1) Datasets

Two datasets with real-world data from location-based social networking platforms are used to verify the algorithms.

**Brightkite [6]:** It contains 4,491,143 check-ins over the period of April 2008–October 2010. In this paper, we used the check-ins of June 2008–September 2008, September 2009–December 2009 and January 2010—April 2010 from Brightkite to construct three types of check-in distributions with different uniformity degree according to the unified longitude and latitude division method, which are, respectively, abbreviated as B1, B2 and B3, and the number of regions is 12.

**Gowalla [6]:** It contains 6,442,890 check-ins over the period of Feburary 2009–October 2010. We used the check-ins of January 2010–April 2010 of Gowalla to construct three types of check-in distributions with different uniformity degree according to different partitioning methods of longitude and latitude, which are, respectively, abbreviated as G1, G2 and G3, and the number of regions is 25, respectively.

**Figure 5:**
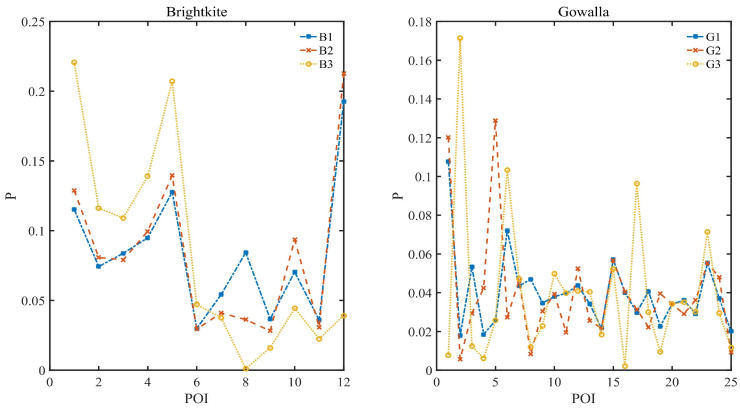
The average data distribution of Brightkite VS. Gowalla.

The average data distribution and the corresponding Gini coefficient of the data are shown in Figure 5 and Table 4, respectively. Therein, Gini coefficient is used to indicate the degree of unevenness of data distribution, which is calculated according to the method of Gini mean difference [52].

Figure 5 and Table 4 both show that the daily check-in data in two datasets fluctuates greatly, meaning a high diveristy. We verify the effectiveness of our algorithms on these real-world datasets in our experiment.

(2) Utility/Privacy Metrics

**Utility Metrics:** The utility uses the maximum relative error as its metrics (see Section 3.3 for details). In this paper, it uses the mean and deviation of the maximum relative error to evaluate the same EDU between KRR and EXP${}_{Q}$ in the dynamic algorithm.

**Privacy Metrics:** The privacy uses two new metrics including the point belief degree and the regional average belief degree (see Defintions 4 and 5 for details). In this paper, it needs to compare the privacy gurantee degree about the expected privacy protection (EPP) under the same expected data utility (EDU) using these two privacy metrics between KRR and EXP${}_{Q}$ in the dynamic algorithm.

(3) Parameter Settings

We evaluate our solutions through experiments using two real-world datasets. The experiments are performed on an Intel Core CPU 2.50-GHz Windows 10 machine equipped with 8 GB of main memory by matlab. In the experiments, the total check-in amount of statistical validity is m=100,000. Three kinds of EDU are η=0.1, 0.08 and 0.05. The expected privacy protection region is $Region\left(\epsilon_{e}\right)=\left[1,1.001,1.002,\cdots ,4\right]$ or $Region\left(\epsilon_{e}\right)=\left[1,1.001,1.002,\cdots ,10\right]$. The modified estimate parameter *w* is set as Table 5. The update threshold parameter is $Re_{thredthold}=0.02$, and the remaining relevant parameters $\epsilon_{0}$ and $\Delta \epsilon_{\eta }$ are set to 0.5 and 0.005, respectively.

### 7.2. Validity and Robustness Evaluation

The performance of validity and robustness of the corresponding dynamic algorithm with KRR and EXP${}_{Q}$ is examined through the dynamic statistics process with two real-world datasets.

**Figure 6:**
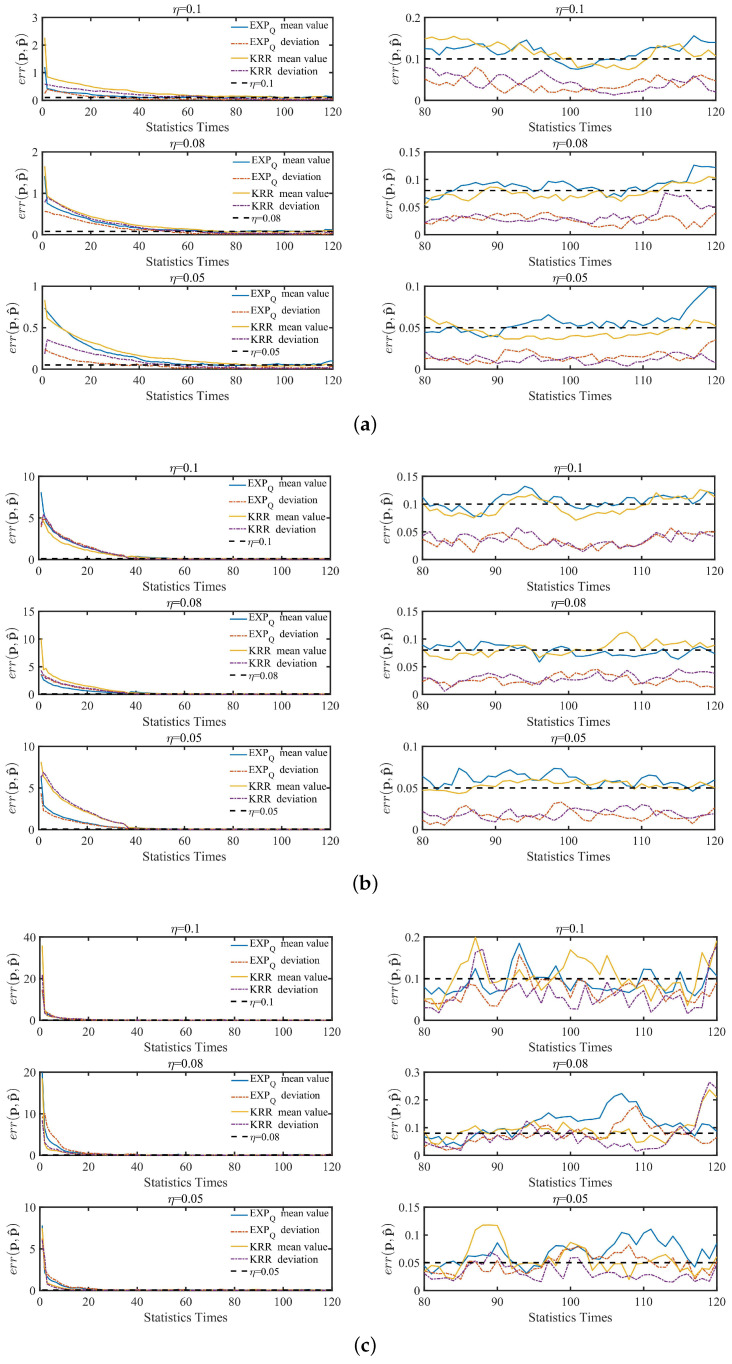
The mean and deviation of $err(p,\hat{p})$ on B1, B2 and B3 subsets of Brightkite. Three η settings of EDU, including 0.1, 0.08 and 0.05, are compared. (**a**) B1; (**b**) B2; (**c**) B3.

**Figure 7:**
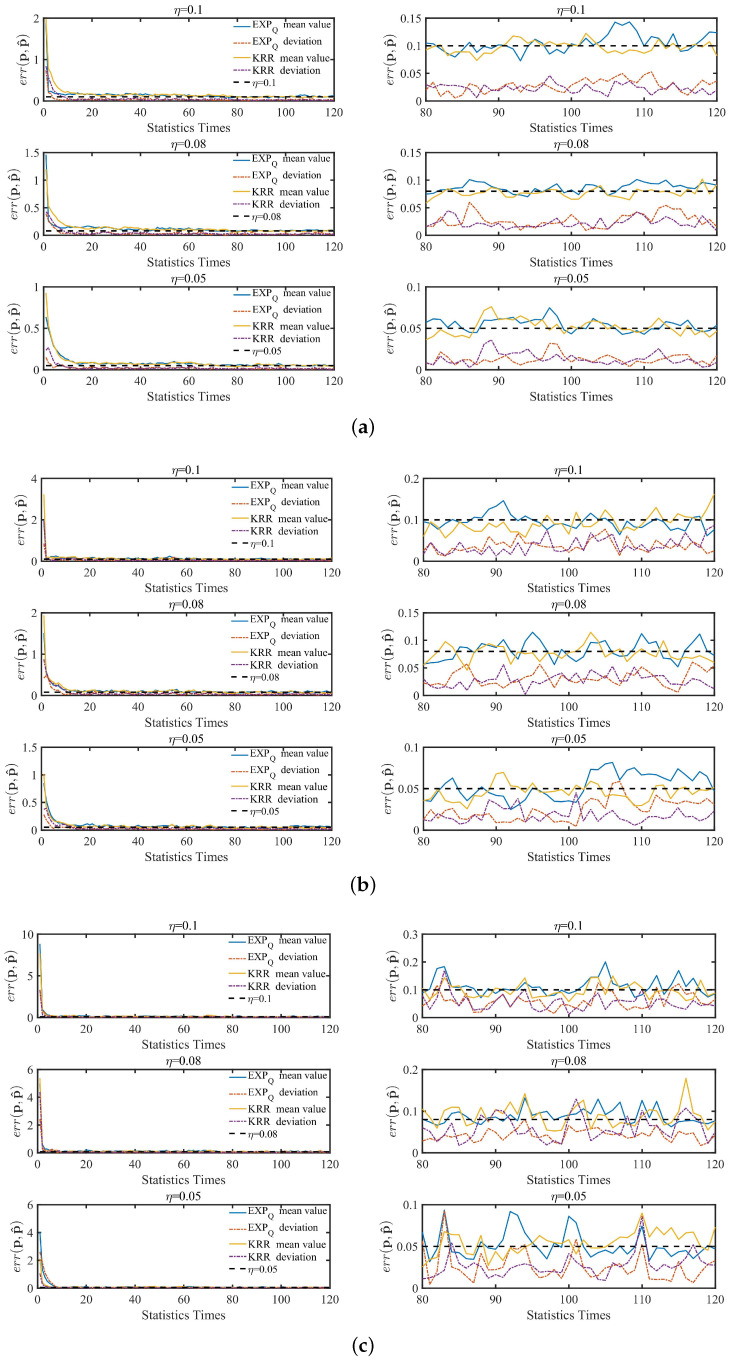
The mean and deviation of $err(p,\hat{p})$ on G1, G2 and G3 subsets of Gowalla. Three η settings of EDU, including 0.1, 0.08 and 0.05, are compared. (**a**) G1; (**b**) G2; (**c**) G3.

Figure 6 and Figure 7 show the mean values and deviations of the maximum relative error $err(p,\hat{p})$ under the three kinds of EDU (η=0.1, 0.08 and 0.05), which are shown by the statistics of the corresponding data subsets under B1, B2, B3, G1, G2 and G3 according to the frequency of once a day. Moreover, the frequency of each day is different and each result is repeated 10 times. In both Figure 6 and Figure 7, the horizontal axis of each graph represents the number of time slices in continuously, and the vertical axis represents the maximum relative error $err\left(p,\hat{p}\right)=max\left(err\left(p_{i},{\hat{p}}_{i}\right)\right)$ between the original data distribution and the estimated data distribution for any i∈[1,n]. As can be seen from the left small graphs of Figure 6 and Figure 7, the corresponding dynamic algorithm with KRR and EXP${}_{Q}$ can converge quickly and maintain the corresponding unified convergence stable state under different data distributions of B1, B2, B3, G1, G2 and G3. This verifies that the dynamic algorithm has a good validity and robustness.

### 7.3. Utility and Privacy Evaluation

The performance of utility and privacy of the corresponding dynamic algorithm with two B-DP mechanisms based on KRR and EXP${}_{Q}$ is also examined through the dynamic statistics process with two real-world datasets. As can also be seen from the right small graphs of Figure 6 and Figure 7, a part of the left small graphs of Figure 6 and Figure 7, it shows clearly that the dynamic algorithm can satisfy the utility even during the dynamic process.

**Figure 8:**
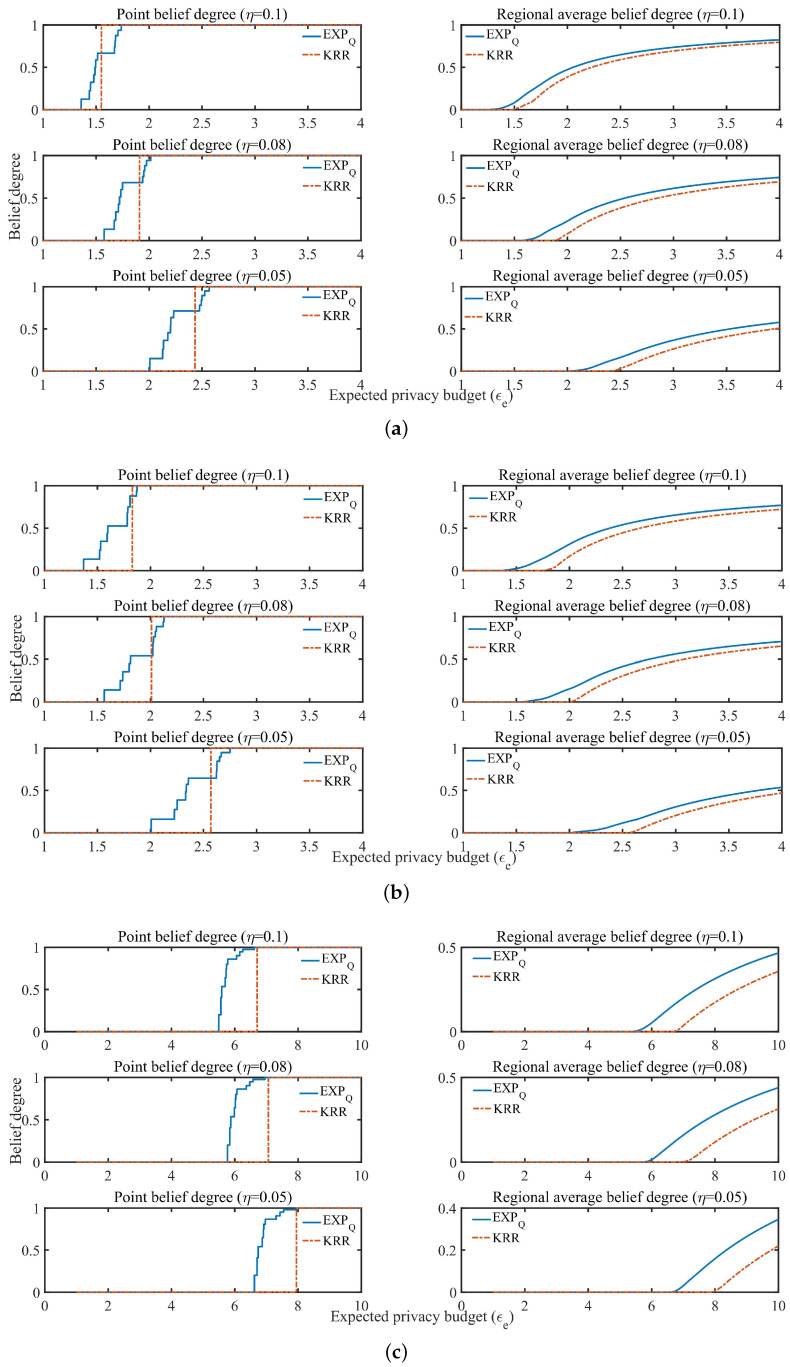
The belief degree on B1, B2 and B3 subsets of Brightkite, where the belief degree includes the point belief degree and the regional average belief degree. (**a**) B1; (**b**) B2; (**c**) B3.

**Figure 9:**
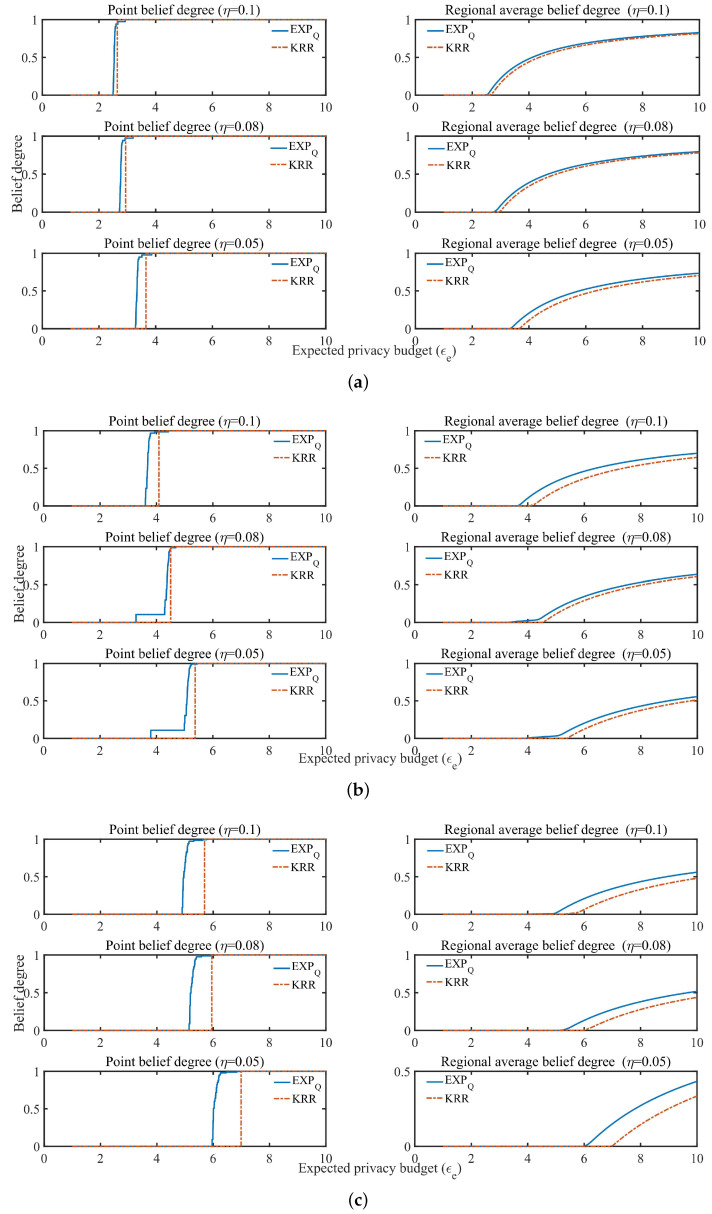
The belief degree on G1, G2 and G3 subsets of Gowalla, where the belief degree includes the point belief degree and the regional average belief degree. (**a**) G1; (**b**) G2; (**c**) G3.

**Figure 10:**
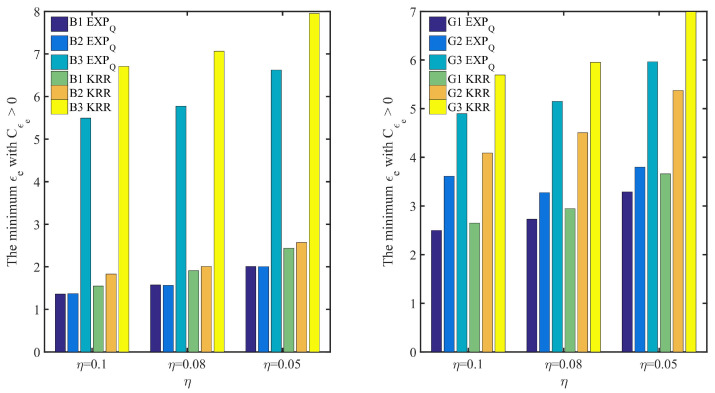
The minimum $\epsilon_{e}$ with $C_{\epsilon_{e}}>0$ based on the same EDU (η) and different data distributions, where $C_{\epsilon_{e}}$ is the point belief degree on the EPP of $\epsilon_{e}$, and moreover, η=0.1, 0.08 and 0.05 represent three kinds of EDU.

**Figure 11:**
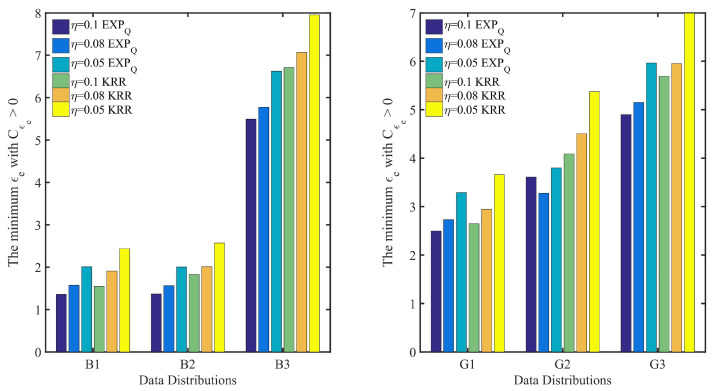
The minimum $\epsilon_{e}$ with $C_{\epsilon_{e}}>0$ based on the same data distribution and different EDU (η), where $C_{\epsilon_{e}}$ is the point belief degree on the EPP of $\epsilon_{e}$, and moreover, η=0.1, 0.08 and 0.05 represent three kinds of EDU.

In addition, Figure 8 and Figure 9 show the point belief degree and the regional average belief degree of each subset of two datasets under the three kinds of EDU (η=0.1, 0.08 and 0.05). In Figure 8 and Figure 9, the horizontal axis of each graph represents the EPP with different expected privacy buget $\epsilon_{e}$ and the vertical axis represents the gurantee degree of the EPP satisfied. It shows that the gurantee degree of the EPP satisfied varies with the data distribution and EDU. For example, from the point belief degree of all small graphs in the left of Figure 8 and Figure 9, the gurantee degree of the EPP satisfied becomes better until its value up to 1 when the expected privacy buget becomes bigger, and the more evener distribution can support the EPP with the smaller $\epsilon_{e}$ to provide a better privacy protection in the same EDU (as Figure 10 shown). The smaller value of EDU, i.e., the lower utility, can generally support the EPP with the smaller $\epsilon_{e}$ to provide a better privacy protection in the same data distribution (as Figure 11 shown).

In Figure 10, for EXP${}_{Q}$ on G1, G2 and G3 with the given EDU (such as η=0.1), it shows clearly that the minimum $\epsilon_{e}$ with $C_{\epsilon_{e}}>0$ is the smallest on G1 and the largest on G3. According to Table 4, G3 is pretty uneven, while G1 is relatively even. It is the same for EXP${}_{Q}$ on B1, B2 and B3, KRR on B1, B2 and B3 as well as on G1, G2 and G3. In Figure 11, for EXP${}_{Q}$ on G1, G2 and G3 with the given data distribution (such as G1), it also shows clearly that the minimum $\epsilon_{e}$ with $C_{\epsilon_{e}}>0$ is the smallest on the EDU with η=0.1 and the largest on the EDU with η=0.05. Similar trends can be observed for EXP${}_{Q}$ on B1, B2 and B3, KRR on B1, B2 and B3 as well as on G1, G2 and G3.

**Figure 12:**
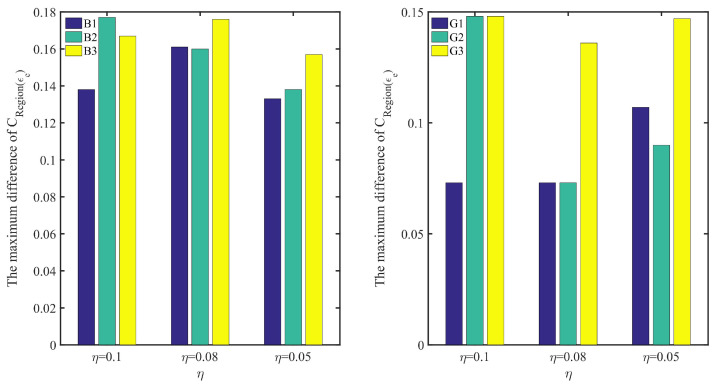
The maximum difference of $C_{\left(Region\left(\epsilon_{e}\right)\right)}$ in EXP${}_{Q}$ minus $C_{\left(Region\left(\epsilon_{e}\right)\right)}$ in KRR with different η, where $C_{\left(Region\left(\epsilon_{e}\right)\right)}$ is the regional average belief degree on the region of $Region\left(\epsilon_{e}\right)=\left[1,1.001,1.002,\cdots ,4\right]$ or $Region\left(\epsilon_{e}\right)=\left[1,1.001,1.002,\cdots ,10\right]$, and η=0.1, 0.08 and 0.05 represents three kinds of EDU.

For the regional average belief degree, the similar results can be concluded from all small graphs in the right of Figure 8 and Figure 9. Moreover, in Figure 12, it shows the maximum difference of $C_{\left(Region\left(\epsilon_{e}\right)\right)}$ in EXP${}_{Q}$ minus $C_{\left(Region\left(\epsilon_{e}\right)\right)}$ in KRR with different η on each subset. It shows that the more unevener the data distribution is, the more bigger the maximum difference is. It means that EXP${}_{Q}$ is more adapt to the unevener data distribution than KRR.

Furthermore, in order to be more objective evaluation of the privacy performance of KRR and EXP${}_{Q}$, it extends to use the privacy metrics of DP to compare the $\epsilon_{\eta }$ on each subset shown in Table 6, where $\epsilon_{\eta }$ refers to the privacy budget of a DP mechanism that just satisfies the EDU η. As can be seen from Table 6, except for η=0.08 and η=0.1 on B3, all the $\epsilon_{\eta }$ of EXP${}_{Q}$ is a little greater than those of KRR, which means that EXP${}_{Q}$ provides a little worse DP than KRR. However, EXP${}_{Q}$ could provide better B-DP from a new perspective of preference for privacy and utility than KRR to provide a good trade-off between them.

## 8. Discussions and Conclusions

This paper proposes a concept of best-effort differential privacy (B-DP) with the expected data utility (EDU) satisfied first and then with the expected privacy protection (EPP) satisfied as much as possible, and designs two new metrics including point belief degree and regional average belief degree to measure the guarantee degree of satisfying the EPP. Moreover, we also provide implementation algorithms, including the corresponding dynamic algorithm of two B-DP mechanisms based on KRR and a newly constructed mechanism EXP${}_{Q}$. Extensive experiments on two real-world check-in datasets verify the effectiveness of the concept of B-DP. It also verifies that the dynamic algorithm has a good validity and robustness, and can satisfy the utility even during the dynamic process. Besides, EXP${}_{Q}$ is more adapt to the unevener data distribution and satisfies a better B-DP than KRR to provide a good trade-off between privacy and utility.

Specifically, the point belief degree measures the guarantee degree of privacy protection for any one expected privacy budget, and the regional average belief degree measures the average guarantee degree of the EPP in a region including multiple expected privacy budgets. Compared with the (ϵ,δ)-DP, the latter can measure only one EPP with the expected privacy budget equal to ϵ and cannot directly measure the average guarantee degree of the EPP, that is, the (ϵ,δ)-DP can only measure the guarantee degree of the EPP when $\epsilon_{e}=\epsilon$, i.e., 1−δ. In addition, many real-world applications can only provide an approximate value of $\epsilon_{e}$ as their EPP, and hence a neighborhood interval with $\epsilon_{e}$ can be regarded as their EPP. Therefore, the regional average belief degree introduced in this paper is very necessary.

Moreover, two B-DP mechanisms based on KRR and newly constructed EXP${}_{Q}$ in this paper are applied to the dynamic collection and publishing of check-in data with relative error as its utility metrics. Therein, KRR itself does not depend on the data distribution, but the dynamic collection and publishing algorithm with B-DP mechanism based on KRR needs to, where the privacy setting parameter has to be adjusted with the influence of data distribution to realize the utility guaranteed firstly in real time. In addition, EXP${}_{Q}$ itself is dependent on the data distribution to realize some of its outputs having strong privacy protection and some having weak privacy protection, which is different from KRR to provide consistent privacy protection intensity. Thus, the dynamic collection and publishing algorithm based on these two B-DP mechanisms needs to depend on the data distribution, and then it has to face the challenges of algorithm validity and robustness with unknown data distribution. Fortunately, the experimental results have already verified that the algorithm can solve both challenges and is promising for the typical application of check-in data.

Besides, if the scenic spots use EXP${}_{Q}$ for privacy protection, the data provider may be more inclined to visit these scenic spots with a large number of visitors, because the regions where these scenic spots are located may have a stronger privacy protection. Compared with the algorithms based on the existing DP mechanisms with consistent privacy protection intensity to realize B-DP, such as KRR in this paper, they maybe do not achieve the EPP at all, but the algorithm based on EXP${}_{Q}$ newly proposed in this paper can achieve the EPP partly at least, that is, EXP${}_{Q}$ can satisfy a better B-DP to provide a good trade-off between privacy and utility.

In a word, although the B-DP dynamic collection and publishing algorithm based on KRR or EXP${}_{Q}$ is not necessarily perfect, it fully proves the feasibility of the concept of B-DP in this paper. It is not only a great step forward for the basic theory of DP, but also provides two feasible solutions for the implementation of DP in practical applications. The two solutions take check-in data as an example, but are not limited to it. They can also be used to other category data for privacy protection where the perturbation model is one input and one output. In the future work, we will make a further discussion on other mechanisms with binary inputs in LDP, where the perturbation model can support one input is perpurbed to multiple outputs, such as RAPPOR, and design them to achieve better B-DP. Moreover, it is an interesting problem about correlated B-DP.

## Figures and Tables

**Table 1:** Comparison of existing literature with the method proposed in this paper.

Selected Papers	Mechanism	Utility First	Privacy First	Privacy Metrics	Utility Metrics	EPP & EDU Involved
Katrina et al. [30]	Laplace	Yes	No	Central DP	Absolute error	No
Liu et al. [23]	Gauss with conditional filtering noise	Yes	No	Central DP	Relative error	Yes, but it may not provide EPP as much as possible
Maryam et al. [31]	Laplace	No	Yes	Central DP	Relative error	No
Xiao et al. [18]	Laplace	No	Yes	Central DP	Relative error	No
Kairouz et al. [25]	W-RR	No	Yes	LDP	KL divergence	No
Erlingsson et al. [28]	RAPPOR	No	Yes	LDP	Standard deviation	No
Bassily et al. [32]	S-Hist	No	Yes	LDP	Absolute error	No
Chen et al. [29]	PCEP	No	Yes	PLDP	KL divergence/relative error	No
Kairouz et al. [24,25]	KRR	No	Yes	LDP	KL divergence	No
Our paper	EXP${}_{Q}$	Yes	No	(Local) B-DP	Relative error	Yes, and it provides EPP as much as possible

**Table 2:** Notations.

Symbol	Description
EDU	Expected data utility
EPP	Expected privacy protection
$\varepsilon_{e}$	Expected privacy budget
*Region*($\epsilon_{e}$)	Expected privacy protection region around $\epsilon_{e}$
η	Expected data utility
$\epsilon_{\eta }$	The privacy budget of a differential privacy mechanism that just meets the expected data utility η
$C_{\epsilon_{e}}$	Point belief degree of $\epsilon_{e}$
$C_{Region(\epsilon_{e})}$	Regional average belief degree of *Region*($\epsilon_{e}$)
p	Original data distribution
$\tilde{p}$	Perturbed data distribution
$\hat{p}$	Estimated data distribution
*S*	Check-in state space
h(S)	Original check-in counts vector
$\tilde{h}\left(S\right)$	Perturbed check-in counts vector
$\hat{h}\left(S\right)$	Estimated check-in counts vector
*Q*	Perturbation probability matrix
$q_{ij}$	The perturbation probability of the original check-in state $S_{j}$ to the check-instate $S_{i}$
KRR	*k*-ary randomized response mechanism
EXP${}_{Q}$	Perturbation mechanism
γ	Privacy setting parameter
$\gamma_{\eta }$	Privacy setting parameter with satisfying the expected data utility η
$\kappa_{n}$	The parameter of privacy protection intensity change point
*w*	Modified estimate parameter
Re${}_{thredthold}$	Update threshold parameter
$err(p,\hat{p})$	The maximum relative error between p and $\hat{p}$

**Table 3:** Gini coefficient VS. Pareto parameter of θ.

Pareto Distribution	θ	Gini Coefficient
P1	1.55	0.4471
P2	1.17	0.3884
P3	0.52	0.2784

**Table 4:** Gini coefficient of data in Brightkite and Gowalla.

Datasets	Data Distributions	Gini Coefficient
Gowalla	G1	0.2357
	G2	0.3488
	G3	0.4465
Brightkite	B1	0.2849
	B2	0.3488
	B3	0.4628

**Table 5:** All kinds of modified estimate parameter *w* used in the dynamic algorithm.

Mechanisms	Brightkite			Gowalla		
	B1	B2	B3	G1	G2	G3
EXP${}_{Q}$	0.045	0.06	0.15	0.18	0.25	0.4
KRR	0.035	0.055	0.15	0.18	0.25	0.4

**Table 6:** $\epsilon_{\eta }$ on each subset, where η=0.1, 0.08 and 0.05 represent three kinds of EDU.

η	Mechanisms	Data Distributions					
		B1	B2	B3	G1	G2	G3
0.1	EXP${}_{Q}$	1.738	1.874	6.627	2.928	4.429	5.774
	KRR	1.55	1.83	6.71	2.65	4.09	5.695
0.08	EXP${}_{Q}$	2.017	2.128	6.967	3.204	4.688	6.072
	KRR	1.91	2.01	7.065	2.945	4.505	5.955
0.05	EXP${}_{Q}$	2.568	2.751	7.995	3.858	5.451	7.041
	KRR	2.435	2.57	7.95	3.66	5.375	6.995

## Data Availability

Two publicly available datasets were analyzed in this study. Both datasets can be found here: http://snap.stanford.edu/data/loc-gowalla.html and http://snap.stanford.edu/data/loc-brightkite.html (accessed on 5 March 2022).

## Appendix A. Proof of Theorem 5

**Proof.** (1) From Definition 11, $\epsilon_{\eta }=max_{1\leq i\leq n}\left(\epsilon_{i}\right)$. According to the definition of LDP, it can be seen that $\epsilon_{\eta }$-EXP${}_{Q}$ satisfies $\epsilon =\epsilon_{\eta }$-LDP. If it wants to proof (2) and (3) of Theorem 5, it needs the following theorems and corollary first. **Theorem** **A1.**
*In perturbation mechanism EXP${}_{Q}$, where i,j∈[1,n] and $\kappa_{n}\in \left[0,n\right]$, it has*

*(a) $q_{jj}\geq q_{ij}$; if $i_{1},i_{2}\in \left[1,n\right]$, $i_{1}\leq i_{2}$ and $i_{1},i_{2}\neq j$, then $q_{i_{1}j}\geq q_{i_{2}j}$.*

*(b) $q_{ii}\geq q_{ij}$; if $j_{1},j_{2}\in \left[1,n\right]$, $j_{1}\leq j_{2}$ and $j_{1},j_{2}\neq i$, then $q_{ij_{1}}\geq q_{ij_{2}}$.*

*(c) for $j_{1}\leq j_{2},$$q_{j_{1}j_{1}}\geq q_{j_{2}j_{2}}$.*

*(d) for $i_{1}\leq i_{2},$${\tilde{p}}_{i_{1}}\geq {\tilde{p}}_{i_{2}}$, where ${\tilde{p}}_{i_{1}},{\tilde{p}}_{i_{2}}$ are the distribution probabilities of $POI_{i_{1}}$ and $POI_{i_{2}}$ after perturbation, respectively.*
**Proof.** See Appendix B. □ **Theorem** **A2.**
*In perturbation mechanism EXP${}_{Q}$, it satisfies the following inequalities, where i∈[1,n].*

*(1) For $\kappa_{n}\in \left[1,n-1\right]$,*

*(i) if $1\leq i\leq \kappa_{n}$ and $j,j^{\prime }\in \left[1,n\right]$, then $e^{-\gamma (1-p_{i+1})}\leq \frac{q_{ij}}{q_{ij^{\prime }}}\leq e^{\gamma (1-p_{i+1})}$;*

*(ii) if $\kappa_{n}<i\leq n-1$, $\kappa_{n}\leq n-2$ and $j,j^{\prime }\in \left[1,n\right]$, then $e^{-\gamma (1+p_{n-i+\kappa_{n}})}\leq \frac{q_{ij}}{q_{ij^{\prime }}}\leq e^{\gamma (1+p_{n-i+\kappa_{n}})}$;*

*(iii) if $\kappa_{n}<i=n$ and $j,j^{\prime }\in \left[1,n\right]$, then $e^{-\gamma (1+p_{\kappa_{n}+1})}\leq \frac{q_{ij}}{q_{ij^{\prime }}}\leq e^{\gamma (1+p_{\kappa_{n}+1})}$.*

*(2) For $\kappa_{n}=0$,*

*(i) if 1≤i≤n−1 and $j,j^{\prime }\in \left[1,n\right]$, then $e^{-\gamma (1+p_{n-i})}\leq \frac{q_{ij}}{q_{ij^{\prime }}}\leq e^{\gamma (1+p_{n-i})}$;*

*(ii) if i=n and $j,j^{\prime }\in \left[1,n\right]$, then $e^{-\gamma (1+p_{1})}\leq \frac{q_{ij}}{q_{ij^{\prime }}}\leq e^{\gamma (1+p_{1})}$.*

*(3) For $\kappa_{n}=n$,*

*(i) if 1≤i≤n−1 and $j,j^{\prime }\in \left[1,n\right]$, then $e^{-\gamma (1-p_{i+1})}\leq \frac{q_{ij}}{q_{ij^{\prime }}}\leq e^{\gamma (1-p_{i+1})}$;*

*(ii) if i=n and $j,j^{\prime }\in \left[1,n\right]$, then $e^{-\gamma (1-p_{n})}\leq \frac{q_{ij}}{q_{ij^{\prime }}}\leq e^{\gamma (1-p_{n})}$.*
**Proof.** See Appendix C. □ From Theorems A1 and A2, it has following Corollary A1. **Corollary** **A1.**
*For any i∈[1,n], let $\epsilon_{i}$ be the actual privacy budget provided by EXP${}_{Q}$ for $POI_{i}$, it has the following properties.*

(1) *For $\kappa_{n}\in \left[1,n-1\right]$,*
  (i) *if $1\leq i\leq \kappa_{n}$, then $\epsilon_{i}=\gamma \left(1-p_{i+1}\right)$;*
  (ii) *if $\kappa_{n}<i\leq n-1$ and $\kappa_{n}\leq n-2$, then $\epsilon_{i}=\gamma \left(1+p_{n-i+\kappa_{n}}\right)$;*
  (iii) *if $\kappa_{n}<i=n$, then $\epsilon_{n}=\gamma \left(1+p_{\kappa_{n}+1}\right)$.*
(2) *For $\kappa_{n}=0$,*
  (i) *if 1≤i≤n−1, then $\epsilon_{i}=\gamma \left(1+p_{n-i}\right)$;*
  (ii) *if i=n, then $\epsilon_{n}=\gamma \left(1+p_{1}\right)$.*
(3) *For $\kappa_{n}=n$,*
  (i) *if 1≤i≤n−1, then $\epsilon_{i}=\gamma \left(1-p_{i+1}\right)$;*
  (ii) *if i=n, then $\epsilon_{n}=\gamma \left(1-p_{n}\right)$.*

Let $\gamma =\gamma_{\eta }$, where $\gamma_{\eta }$ is the privacy setting parameter γ of EXP${}_{Q}$ that satisfies the EDU $err(p,\hat{p})=\eta$. According to Theorem A2, the actual privacy budget for each $POI_{i}$ can be set to $\epsilon_{i}=\varphi \left(\gamma_{\eta },p\right)$, which is a function of $\gamma_{\eta }$ and p. It is easy to obtain that $\epsilon_{i}$ is a monotonic non-decreasing function according to Theorem A2 and Corollary A1 for a fixed $\kappa_{n}$. Therefore, the $\epsilon_{\eta }=max_{1\leq i\leq n}\left(\epsilon_{i}\right)$-EXP${}_{Q}$ satisfies (2) and (3) of Theorem 5 that can be proved as follows. (2) When $\kappa_{n}$ is fixed and the point belief degree of $\epsilon_{\eta }$-EXP${}_{Q}$ is $C_{\epsilon_{e}}=\sum_{i=1}^{n}{\tilde{p}}_{i}\chi \left(\epsilon_{i},\epsilon_{e}\right)$, if $C_{\epsilon_{e}}$ is maximized, then $\epsilon_{\eta }$-EXP${}_{Q}$ satisfies $\left(\epsilon_{\eta },C_{\epsilon_{e}}\right)$-Best-B-DP. According to Corollary A1, it can be known that the larger $p_{i}$ is, the smaller $\epsilon_{i}$ is, indicating that in the same $\epsilon_{e}$, $\epsilon_{i}$ can satisfy $\epsilon_{e}$ at first, and the proportion that it satisfies is ${\tilde{p}}_{i}$. According to Theorem A2, when $p_{i}$ is larger, ${\tilde{p}}_{i}$ will always be larger too. Hence, $C_{\epsilon_{e}}$ is also maximized under a fixed $\kappa_{n}$. (3) According to Definition 10 and Corollary A1, in EXP${}_{Q}$, the data distribution p satisfies $p_{1}\geq \cdots \geq p_{i}\geq \cdots \geq p_{n}$ and the actual privacy budget satisfies $\epsilon_{1}\leq \cdots \leq \epsilon_{i}\leq \cdots \leq \epsilon_{n}$. Suppose $\epsilon_{i}\leq \epsilon_{e}<\epsilon_{i+1}$, then the probability of satisfying the EPP $\epsilon_{e}$ under the EDU η is $C_{\epsilon_{e}}=\sum_{j=1}^{n}{\tilde{p}}_{j}\chi \left(\epsilon_{j},\epsilon_{e}\right)=\sum_{j=1}^{i}{\tilde{p}}_{j}.$ Let R1={1,2,⋯,i},R2={i+1,i+2,⋯,n}, then $P\left(k\in R1\right)=C_{\epsilon_{e}},P\left(k\in R2\right)=1-C_{\epsilon_{e}}$. Hence, For any user *u* and $k,j^{\prime },j\in \left[1,n\right]$, it has

(A1) $$\begin{array}{cc} \; & P(|ln\left(\frac{q_{kj^{\prime }}}{q_{kj}}\right)|\leq \epsilon_{e})\\ \; & =P(|ln\left(\frac{q_{kj^{\prime }}}{q_{kj}}\right)|\leq \epsilon_{e}\left|k\in R1\right)P\left(k\in R1\right)\\ \; & +P(|ln\left(\frac{q_{kj^{\prime }}}{q_{kj}}\right)|\leq \epsilon_{e}\left|k\in R2\right)P\left(k\in R2\right)\\ \; & =1\cdot C_{\epsilon_{e}}+0\cdot \left(1-C_{\epsilon_{e}}\right)\\ \; & =C_{\epsilon_{e}}. \end{array}$$

Therefore, it is easy to obtain

(A2) $$\begin{array}{c} q_{kj^{\prime }}\leq e^{\epsilon_{e}}q_{kj}+\left(1-C_{\epsilon_{e}}\right) \end{array}$$

for any user *u* and $k,j^{\prime },j\in \left[1,n\right]$. Therefore, according to Definitions 2, 3 and Theorem A2, $\epsilon_{\eta }$-EXP${}_{Q}$ with the point belief degree $C_{\epsilon_{e}}$ satisfies $\left(\epsilon_{e},1-C_{\epsilon_{e}}\right)$-LDP.

## Appendix B. Proof of A1

**Proof.** Because of the perturbation mechanism EXP${}_{Q}$, the check-in data distribution $p={\left[p_{1},p_{2},\cdots ,p_{n}\right]}^{T}$ satisfies $p_{1}\geq p_{2}\geq \cdots \geq p_{n}$. In accordance with the $\kappa_{n}\in \left[1,n-1\right],\kappa_{n}=n$ and $\kappa_{n}=0$, it can be discussed for three cases. For $\kappa_{n}\in \left[1,n-1\right]$, if the perturbation mechanism EXP${}_{Q}$ satisfies Theorem A1, then the other two cases, $\kappa_{n}=n$ and $\kappa_{n}=0$, are also proved by using the same method. For $\kappa_{n}\in \left[1,n-1\right]$, it can be discussed as follows based on Theorem 4. (a) For any i∈[1,n], the probability $q_{ij}$ that the check-in state of $POI_{j}$ is perturbed to that of $POI_{i}$ satisfies the following cases. (i) For $i\leq \kappa_{n}$ and i≠j, it has $q_{jj}=\frac{1}{\Omega_{j}}\geq q_{ij}=\frac{e^{-\gamma (1-p_{i})}}{\Omega_{j}}$. Moreover, for $i_{1}\leq i_{2}$, $i_{1},i_{2}\neq j$ and $i_{1},i_{2}\leq \kappa_{n}$, it has $q_{i_{1}j}=\frac{e^{-\gamma (1-p_{i_{1}})}}{\Omega_{j}}\geq q_{i_{2}j}=\frac{e^{-\gamma (1-p_{i_{2}})}}{\Omega_{j}}$. (ii) For $i>\kappa_{n}$ and i≠j, it has $q_{jj}=\frac{1}{\Omega_{j}}\geq q_{ij}=\frac{e^{-\gamma (1+p_{n-i+\kappa_{n}+1})}}{\Omega_{j}}$. Moreover, for $i_{1},i_{2}>\kappa_{n}$, $i_{1}\leq i_{2}$, and $i_{1},i_{2}\neq j$, it has $q_{i_{1}j}=\frac{e^{-\gamma (1+p_{n-i_{1}+\kappa_{n}+1})}}{\Omega_{j}}\geq q_{i_{2}j}=\frac{e^{-\gamma (1+p_{n-i_{2}+\kappa_{n}+1})}}{\Omega_{j}}$. (iii) For $i_{1}\neq j$, $i_{1}\leq \kappa_{n}$, $i_{2}\neq j$ and $i_{2}>\kappa_{n}$, it has $q_{i_{1}j}=\frac{e^{-\gamma (1-p_{i_{1}})}}{\Omega_{j}}\geq q_{i_{2}j}=\frac{e^{-\gamma (1+p_{n-i_{2}+\kappa_{n}+1})}}{\Omega_{j}}$. From the above discussions (*i*–*iii*), the property (a) in this theorem holds for $i_{1},i_{2}\neq j$ and $\kappa_{n}\in \left[1,n-1\right]$. (b.1) For j∈[1,n], the probability $q_{ij}$ that the check-in state of $POI_{j}$ is perturbed to that of $POI_{i}$ satisfies the following cases. (i) For $i,j\leq \kappa_{n}$ and j≠i, it has

(A3) $$\begin{array}{cc} q_{ii} & =\frac{1}{\Omega_{i}}=\frac{e^{-\gamma (1-p_{i})}}{e^{-\gamma (1-p_{i})}\Omega_{i}}\\ \; & =\frac{e^{-\gamma (1-p_{i})}}{e^{-\gamma (1-p_{i})}\left(1+\sum_{k=1,k\neq i}^{\kappa_{n}}e^{-\gamma (1-p_{k})}+\sum_{k=\kappa_{n}+1}^{n}e^{-\gamma (1+p_{n-k+\kappa_{n}+1})}\right)}\\ \; & =\frac{e^{-\gamma (1-p_{i})}}{e^{-\gamma (1-p_{i})}\left(1+e^{-\gamma (1-p_{j})}+\sum_{k=1,k\neq i,k\neq j}^{\kappa_{n}}e^{-\gamma (1-p_{k})}+\sum_{k=\kappa_{n}+1}^{n}e^{-\gamma (1+p_{n-k+\kappa_{n}+1})}\right)}\\ \; & \geq \frac{e^{-\gamma (1-p_{i})}}{1+\sum_{k=1,k\neq j}^{\kappa_{n}}e^{-\gamma (1-p_{k})}+\sum_{k=\kappa_{n}+1}^{n}e^{-\gamma (1+p_{n-k+\kappa_{n}+1})}}=\frac{e^{-\gamma (1-p_{i})}}{\Omega_{j}}=q_{ij}. \end{array}$$

(ii) For j≠i, $i\leq \kappa_{n}$ and $j>\kappa_{n}$, it has

(A4) $$\begin{array}{cc} q_{ii} & =\frac{1}{\Omega_{i}}=\frac{e^{-\gamma (1-p_{i})}}{e^{-\gamma (1-p_{i})}\Omega_{i}}\\ \; & =\frac{e^{-\gamma (1-p_{i})}}{e^{-\gamma (1-p_{i})}\left(1+\sum_{k=1,k\neq i}^{\kappa_{n}}e^{-\gamma (1-p_{k})}+\sum_{k=\kappa_{n}+1}^{n}e^{-\gamma (1+p_{n-k+\kappa_{n}+1})}\right)}\\ \; & =\frac{e^{-\gamma (1-p_{i})}}{e^{-\gamma (1-p_{i})}\left(1+e^{-\gamma (1+p_{n-j+\kappa_{n}+1})}+\sum_{k=1,k\neq i}^{\kappa_{n}}e^{-\gamma (1-p_{k})}+\sum_{k=\kappa_{n}+1,k\neq j}^{n}e^{-\gamma (1+p_{n-k+\kappa_{n}+1})}\right)}\\ \; & \geq \frac{e^{-\gamma (1-p_{i})}}{e^{-\gamma (2-p_{i}+p_{n-j+\kappa_{n}+1})}+\sum_{k=1}^{\kappa_{n}}e^{-\gamma (1-p_{k})}+\sum_{k=\kappa_{n}+1,k\neq j}^{n}e^{-\gamma (1+p_{n-k+\kappa_{n}+1})}}\\ \; & \geq \frac{e^{-\gamma (1-p_{i})}}{1+\sum_{k=1}^{\kappa_{n}}e^{-\gamma (1-p_{k})}+\sum_{k=\kappa_{n}+1,k\neq j}^{n}e^{-\gamma (1+p_{n-k+\kappa_{n}+1})}}=\frac{e^{-\gamma (1-p_{i})}}{\Omega_{j}}=q_{ij}. \end{array}$$

(iii) For j≠i, $i>\kappa_{n}$ and $j\leq \kappa_{n}$, it has

(A5) $$\begin{array}{cc} q_{ii} & =\frac{1}{\Omega_{i}}=\frac{e^{-\gamma (1+p_{n-i+\kappa_{n}+1})}}{e^{-\gamma (1+p_{n-i+\kappa_{n}+1})}\Omega_{i}}\\ \; & =\frac{e^{-\gamma (1+p_{n-i+\kappa_{n}+1})}}{e^{-\gamma (1+p_{n-i+\kappa_{n}+1})}\left(1+\sum_{k=1}^{\kappa_{n}}e^{-\gamma (1-p_{k})}+\sum_{k=\kappa_{n}+1,k\neq i}^{n}e^{-\gamma (1+p_{n-k+\kappa_{n}+1})}\right)}\\ \; & =\frac{e^{-\gamma (1+p_{n-i+\kappa_{n}+1})}}{e^{-\gamma (1+p_{n-i+\kappa_{n}+1})}\left(1+e^{-\gamma (1-p_{j})}+\sum_{k=1,k\neq j}^{\kappa_{n}}e^{-\gamma (1-p_{k})}+\sum_{k=\kappa_{n}+1,k\neq i}^{n}e^{-\gamma (1+p_{n-k+\kappa_{n}+1})}\right)}\\ \; & \geq \frac{e^{-\gamma (1+p_{n-i+\kappa_{n}+1})}}{e^{-\gamma (2+p_{n-i+\kappa_{n}+1}-p_{j})}+\sum_{k=1,k\neq j}^{\kappa_{n}}e^{-\gamma (1-p_{k})}+\sum_{k=\kappa_{n}+1}^{n}e^{-\gamma (1+p_{n-k+\kappa_{n}+1})}}\\ \; & \geq \frac{e^{-\gamma (1+p_{n-i+\kappa_{n}+1})}}{1+\sum_{k=1,k\neq j}^{\kappa_{n}}e^{-\gamma (1-p_{k})}+\sum_{k=\kappa_{n}+1}^{n}e^{-\gamma (1+p_{n-k+\kappa_{n}+1})}}\\ & =\frac{e^{-\gamma (1+p_{n-i+\kappa_{n}+1})}}{\Omega_{j}}\\ & =q_{ij}. \end{array}$$

(iv) For j≠i, $i>\kappa_{n}$ and $j>\kappa_{n}$, it has

(A6) $$\begin{array}{cc} q_{ii} & =\frac{1}{\Omega_{i}}\\ \; & =\frac{e^{-\gamma (1+p_{n-i+\kappa_{n}+1})}}{e^{-\gamma (1+p_{n-i+\kappa_{n}+1})}\Omega_{i}}\\ \; & =\frac{e^{-\gamma (1+p_{n-i+\kappa_{n}+1})}}{e^{-\gamma (1+p_{n-i+\kappa_{n}+1})}\left(1+\sum_{k=1}^{\kappa_{n}}e^{-\gamma (1-p_{k})}+\sum_{k=\kappa_{n}+1,k\neq i}^{n}e^{-\gamma (1+p_{n-k+\kappa_{n}+1})}\right)}\\ \; & \geq \frac{e^{-\gamma (1+p_{n-i+\kappa_{n}+1})}}{e^{-\gamma (2+p_{n-i+\kappa_{n}+1}+p_{n-j+\kappa_{n}+1})}+\sum_{k=1}^{\kappa_{n}}e^{-\gamma (1-p_{k})}+\sum_{k=\kappa_{n}+1,k\neq j}^{n}e^{-\gamma (1+p_{n-k+\kappa_{n}+1})}}\\ \; & \geq \frac{e^{-\gamma (1+p_{n-i+\kappa_{n}+1})}}{1+\sum_{k=1}^{\kappa_{n}}e^{-\gamma (1-p_{k})}+\sum_{k=\kappa_{n}+1,k\neq j}^{n}e^{-\gamma (1+p_{n-k+\kappa_{n}+1})}}=\frac{e^{-\gamma (1+p_{n-i+\kappa_{n}+1})}}{\Omega_{j}}=q_{ij}. \end{array}$$

From the above discussions (*i*–*iv*), it can be seen that $q_{ii}\geq q_{ij}$ for j∈[1,n]. (b.2) For $j_{1}\leq j_{2}$, $j_{1},j_{2}\neq i$ and $j_{1},j_{2}\in \left[1,n\right]$, the probability $q_{ij_{1}}$ that the check-in state of $POI_{j_{1}}$ is perturbed to that of $POI_{i}$ and the probability $q_{ij_{2}}$ that the check-in state of $POI_{j_{2}}$ is perturbed to that of $POI_{i}$ satisfy the following cases. (i) For $i\leq \kappa_{n}$ and $j_{1},j_{2}\leq \kappa_{n}$, it has

(A7) $$\begin{array}{cc} q_{ij_{1}} & =\frac{e^{-\gamma (1-p_{i})}}{\Omega_{j_{1}}}\\ \; & =\frac{e^{-\gamma (1-p_{i})}}{1+\sum_{k=1,k\neq j_{1}}^{\kappa_{n}}e^{-\gamma (1-p_{k})}+\sum_{k=\kappa_{n}+1}^{n}e^{-\gamma (1+p_{n-k+\kappa_{n}+1})}}\\ \; & =\frac{e^{-\gamma (1-p_{i})}}{1+e^{-\gamma (1-p_{j_{2}})}+\sum_{k=1,k\neq j_{1},k\neq j_{2}}^{\kappa_{n}}e^{-\gamma (1-p_{k})}+\sum_{k=\kappa_{n}+1}^{n}e^{-\gamma (1+p_{n-k+\kappa_{n}+1})}}\\ \; & \geq \frac{e^{-\gamma (1-p_{i})}}{1+e^{-\gamma (1-p_{j_{1}})}+\sum_{k=1,k\neq j_{1},k\neq j_{2}}^{\kappa_{n}}e^{-\gamma (1-p_{k})}+\sum_{k=\kappa_{n}+1}^{n}e^{-\gamma (1+p_{n-k+\kappa_{n}+1})}}\\ \; & =\frac{e^{-\gamma (1-p_{i})}}{1+\sum_{k=1,k\neq j_{2}}^{\kappa_{n}}e^{-\gamma (1-p_{k})}+\sum_{k=\kappa_{n}+1}^{n}e^{-\gamma (1+p_{n-k+\kappa_{n}+1})}}=\frac{e^{-\gamma (1-p_{i})}}{\Omega_{j_{2}}}=q_{ij_{2}}. \end{array}$$

(ii) For $j_{1},j_{2}>\kappa_{n}$ and $i\leq \kappa_{n}$, it has

(A8) $$\begin{array}{cc} q_{ij_{1}} & =\frac{e^{-\gamma (1-p_{i})}}{\Omega_{j_{1}}}\\ \; & =\frac{e^{-\gamma (1-p_{i})}}{1+\sum_{k=1}^{\kappa_{n}}e^{-\gamma (1-p_{k})}+\sum_{k=\kappa_{n}+1,k\neq j_{1}}^{n}e^{-\gamma (1+p_{n-k+\kappa_{n}+1})}}\\ \; & =\frac{e^{-\gamma (1-p_{i})}}{1+e^{-\gamma (1+p_{n-j_{2}+\kappa_{n}+1})}+\sum_{k=1}^{\kappa_{n}}e^{-\gamma (1-p_{k})}+\sum_{k=\kappa_{n}+1,k\neq j_{1},k\neq j_{2}}^{n}e^{-\gamma (1+p_{n-k+\kappa_{n}+1})}}\\ \; & \geq \frac{e^{-\gamma (1-p_{i})}}{1+e^{-\gamma (1+p_{n-j_{1}+\kappa_{n}+1})}+\sum_{k=1}^{\kappa_{n}}e^{-\gamma (1-p_{k})}+\sum_{k=\kappa_{n}+1,k\neq j_{1},k\neq j_{2}}^{n}e^{-\gamma (1+p_{n-k+\kappa_{n}+1})}}\\ \; & =\frac{e^{-\gamma (1-p_{i})}}{1+\sum_{k=1}^{\kappa_{n}}e^{-\gamma (1-p_{k})}+\sum_{k=\kappa_{n}+1,k\neq j_{2}}^{n}e^{-\gamma (1+p_{n-k+\kappa_{n}+1})}}=\frac{e^{-\gamma (1-p_{i})}}{\Omega_{j_{2}}}=q_{ij_{2}}. \end{array}$$

(iii) For $i\leq \kappa_{n}$, $j_{1}\leq \kappa_{n}$ and $j_{2}>\kappa_{n}$, it has

(A9) $$\begin{array}{cc} q_{ij_{1}} & =\frac{e^{-\gamma (1-p_{i})}}{\Omega_{j_{1}}}\\ \; & =\frac{e^{-\gamma (1-p_{i})}}{1+\sum_{k=1,k\neq j_{1}}^{\kappa_{n}}e^{-\gamma (1-p_{k})}+\sum_{k=\kappa_{n}+1}^{n}e^{-\gamma (1+p_{n-k+\kappa_{n}+1})}}\\ \; & =\frac{e^{-\gamma (1-p_{i})}}{1+e^{-\gamma (1+p_{n-j_{2}+\kappa_{n}+1})}+\sum_{k=1,k\neq j_{1}}^{\kappa_{n}}e^{-\gamma (1-p_{k})}+\sum_{k=\kappa_{n}+1,k\neq j_{2}}^{n}e^{-\gamma (1+p_{n-k+\kappa_{n}+1})}}\\ \; & \geq \frac{e^{-\gamma (1-p_{i})}}{1+e^{-\gamma (1-p_{j_{1}})}+\sum_{k=1,k\neq j_{1}}^{\kappa_{n}}e^{-\gamma (1-p_{k})}+\sum_{k=\kappa_{n}+1,k\neq j_{2}}^{n}e^{-\gamma (1+p_{n-k+\kappa_{n}+1})}}\\ \; & =\frac{e^{-\gamma (1-p_{i})}}{1+\sum_{k=1}^{\kappa_{n}}e^{-\gamma (1-p_{k})}+\sum_{k=\kappa_{n}+1,k\neq j_{2}}^{n}e^{-\gamma (1+p_{n-k+\kappa_{n}+1})}}=\frac{e^{-\gamma (1-p_{i})}}{\Omega_{j_{2}}}=q_{ij_{2}}. \end{array}$$

From the above discussions (*i*–*iii*) of (b.2), when $i\leq \kappa_{n}$, for $j_{1},j_{2}\neq i$, $j_{1}\leq j_{2}$ and $j_{1},j_{2}\in \left[1,n\right]$, $q_{ij_{1}}\geq q_{ij_{2}}$. When $i>\kappa_{n}$, for $j_{1},j_{2}\neq i$, $j_{1}\leq j_{2}$ and $j_{1},j_{2}\in \left[1,n\right]$, we can use the similar method to get $q_{ij_{1}}\geq q_{ij_{2}}$. Then it has $q_{ij_{1}}\geq q_{ij_{2}}$ for $i,j_{1},j_{2}\in \left[1,n\right]$, $j_{1}\leq j_{2}$ and $j_{1},j_{2}\neq i$. Hence, from (b.1∼b.2), when $\kappa_{n}\in \left[1,n-1\right]$, the part (b) of Theorem A1 holds. (c) According to the property (b) and its proof process, it is easy to get $\frac{1}{\Omega_{j_{1}}}\geq \frac{1}{\Omega_{j_{2}}}$, i.e., $q_{j_{1}j_{1}}\geq q_{j_{2}j_{2}}$ holds for $j_{1}\leq j_{2}$ and $j_{1},j_{2}\neq i$. (d) Since ${\tilde{p}}_{i}=\sum_{j=1}^{n}q_{ij}p_{j}$ for i∈[1,n], it has $i_{1}\leq i_{2}$ that implies $p_{i_{1}}\geq p_{i_{2}}$, and it has Formula (A10). According the property (a)$q_{i_{1}j}\geq q_{i_{2}j}$ and Formula (A10), it can be seen that $\sum_{j=1,j\neq i_{1},j\neq i_{2}}^{n}\left(q_{i_{1}j}-q_{i_{2}j}\right)p_{j}\geq 0$.

(A10) $$\begin{array}{cc} {\tilde{p}}_{i_{1}}-{\tilde{p}}_{i_{2}} & =\sum_{j=1}^{n}q_{i_{1}j}p_{j}-\sum_{j=1}^{n}q_{i_{2}j}p_{j}\\ \; & =\sum_{j=1,j\neq i_{1},j\neq i_{2}}^{n}\left(q_{i_{1}j}-q_{i_{2}j}\right)p_{j}+q_{i_{1}i_{1}}p_{i_{1}}+q_{i_{1}i_{2}}p_{i_{2}}-q_{i_{2}i_{1}}p_{i_{1}}-q_{i_{2}i_{2}}p_{i_{2}}\\ \; & =\sum_{j=1,j\neq i_{1},j\neq i_{2}}^{n}\left(q_{i_{1}j}-q_{i_{2}j}\right)p_{j}+\left(q_{i_{1}i_{1}}-q_{i_{2}i_{1}}\right)p_{i_{1}}-\left(q_{i_{2}i_{2}}-q_{i_{1}i_{2}}\right)p_{i_{2}}. \end{array}$$

If it wants to prove ${\tilde{p}}_{i_{1}}\geq {\tilde{p}}_{i_{2}}$ always stands up for $i_{1}\leq i_{2}$, then it just has to prove $\left(q_{i_{1}i_{1}}-q_{i_{2}i_{1}}\right)p_{i_{1}}-\left(q_{i_{2}i_{2}}-q_{i_{1}i_{2}}\right)p_{i_{2}}\geq 0$. Similarly, it only wants to discuss the following case $\kappa_{n}\in \left[1,n-1\right]$, and the other two cases, that is, $\kappa_{n}=0$ and $\kappa_{n}=n$ can also be proved by using the same method. (i) For $1\leq i_{1}\leq i_{2}\leq \kappa_{n}$, it has

(A11) $$\begin{array}{cc} \; & \left(q_{i_{1}i_{1}}-q_{i_{2}i_{1}}\right)p_{i_{1}}-\left(q_{i_{2}i_{2}}-q_{i_{1}i_{2}}\right)p_{i_{2}}\\ \; & =\frac{1-e^{-\gamma (1-p_{i_{2}})}}{\Omega_{i_{1}}}p_{i_{1}}-\frac{1-e^{-\gamma (1-p_{i_{1}})}}{\Omega_{i_{2}}}p_{i_{2}}\\ \; & \geq \frac{1-e^{-\gamma (1-p_{i_{1}})}}{\Omega_{i_{2}}}p_{i_{1}}-\frac{1-e^{-\gamma (1-p_{i_{1}})}}{\Omega_{i_{2}}}p_{i_{2}}\\ \; & =\frac{1-e^{-\gamma (1-p_{i_{1}})}}{\Omega_{i_{2}}}\left(p_{i_{1}}-p_{i_{2}}\right)\geq 0. \end{array}$$

(ii) For $1\leq i_{1}\leq \kappa_{n}$ and $n\geq i_{2}>\kappa_{n}$, it can also obtain

(A12) $$\begin{array}{cc} \; & \left(q_{i_{1}i_{1}}-q_{i_{2}i_{1}}\right)p_{i_{1}}-\left(q_{i_{2}i_{2}}-q_{i_{1}i_{2}}\right)p_{i_{2}}\\ \; & =\frac{1-e^{-\gamma (1+p_{n-i_{2}+\kappa_{n}+1})}}{\Omega_{i_{1}}}p_{i_{1}}-\frac{1-e^{-\gamma (1-p_{i_{1}})}}{\Omega_{i_{2}}}p_{i_{2}}\\ \; & \geq \frac{1-e^{-\gamma (1-p_{i_{2}})}}{\Omega_{i_{1}}}p_{i_{1}}-\frac{1-e^{-\gamma (1-p_{i_{1}})}}{\Omega_{i_{2}}}p_{i_{2}}\\ \; & \geq \frac{1-e^{-\gamma (1-p_{i_{2}})}}{\Omega_{i_{2}}}p_{i_{1}}-\frac{1-e^{-\gamma (1-p_{i_{1}})}}{\Omega_{i_{2}}}p_{i_{2}}\\ \; & \geq \frac{1-e^{-\gamma (1-p_{i_{1}})}}{\Omega_{i_{2}}}p_{i_{1}}-\frac{1-e^{-\gamma (1-p_{i_{1}})}}{\Omega_{i_{2}}}p_{i_{2}}\\ \; & =\frac{1-e^{-\gamma (1-p_{i_{1}})}}{\Omega_{i_{2}}}\left(p_{i_{1}}-p_{i_{2}}\right)\geq 0. \end{array}$$

(iii) For $\kappa_{n}<i_{1}\leq i_{2}\leq n$, it can also get

(A13) $$\begin{array}{cc} \; & \left(q_{i_{1}i_{1}}-q_{i_{2}i_{1}}\right)p_{i_{1}}-\left(q_{i_{2}i_{2}}-q_{i_{1}i_{2}}\right)p_{i_{2}}\\ \; & =\frac{1-e^{-\gamma (1+p_{n-i_{2}+\kappa_{n}+1})}}{\Omega_{i_{1}}}p_{i_{1}}-\frac{1-e^{-\gamma (1+p_{n-i_{1}+\kappa_{n}+1})}}{\Omega_{i_{2}}}p_{i_{2}}\\ \; & \geq \frac{1-e^{-\gamma (1+p_{n-i_{1}+\kappa_{n}+1})}}{\Omega_{i_{1}}}p_{i_{1}}-\frac{1-e^{-\gamma (1+p_{n-i_{1}+\kappa_{n}+1})}}{\Omega_{i_{2}}}p_{i_{2}}\\ \; & \geq \frac{1-e^{-\gamma (1+p_{n-i_{1}+\kappa_{n}+1})}}{\Omega_{i_{2}}}p_{i_{1}}-\frac{1-e^{-\gamma (1+p_{n-i_{1}+\kappa_{n}+1})}}{\Omega_{i_{2}}}p_{i_{2}}\\ \; & =\frac{1-e^{-\gamma (1+p_{n-i_{1}+\kappa_{n}+1})}}{\Omega_{i_{2}}}\left(p_{i_{1}}-p_{i_{2}}\right)\geq 0, \end{array}$$

From the above discussions (i∼iii), the part (d) in Theorem A1 is true. Therefore, from (a∼d), the result follows. □

## Appendix C. Proof of Theorem A2

**Proof.** It is only to deal with case (1) here, and a similar statement can be made of cases (2) or (3). From Theorem A1, it can be seen that $q_{ii}\geq q_{ij}$ for i,j∈[1,n] and it has $q_{ij_{1}}\geq q_{ij_{2}}$ for $j_{1}\leq j_{2}$, $j_{1},j_{2}\in \left[1,n\right]$ and $j_{1},j_{2}\neq i$. Hence, it has the following cases to discuss for $\kappa_{n}\in \left[1,n-1\right]$. (i) If $i\leq \kappa_{n}$ and $j,j^{\prime }\in \left[1,n\right]$, then it has Formula (A14).

(A14) $$\begin{array}{cc} \; & \frac{q_{ij}}{q_{ij^{\prime }}}\leq \frac{q_{ii}}{q_{in}}=\frac{\frac{1}{\Omega_{i}}}{\frac{e^{-\gamma (1-p_{i})}}{\Omega_{n}}}\\ \; & =e^{\gamma (1-p_{i})}\frac{\Omega_{n}}{\Omega_{i}}\\ \; & =e^{\gamma (1-p_{i})}\frac{1+e^{-\gamma (1-p_{i})}+\sum_{k=1,k\neq i}^{\kappa_{n}}e^{-\gamma (1-p_{k})}+\sum_{k=\kappa_{n}+1,k\neq n}^{n}e^{-\gamma (1+p_{n-k+\kappa_{n}+1})}}{1+e^{-\gamma (1+p_{\kappa_{n}+1})}+\sum_{k=1,k\neq i}^{\kappa_{n}}e^{-\gamma (1-p_{k})}+\sum_{k=\kappa_{n}+1,k\neq n}^{n}e^{-\gamma (1+p_{n-k+\kappa_{n}+1})}}\\ \; & \leq e^{\gamma (1-p_{i+1})}\\ \; & \times \frac{1+e^{-\gamma (1-p_{i})}+\sum_{k=1,k\neq i}^{\kappa_{n}}e^{-\gamma (1-p_{k})}+\sum_{k=\kappa_{n}+1,k\neq n}^{n}e^{-\gamma (1+p_{n-k+\kappa_{n}+1})}}{e^{\gamma (p_{i}-p_{i+1})}+e^{\gamma (p_{i}-p_{i+1})}e^{-\gamma (1+p_{\kappa_{n}+1})}+\sum_{k=1,k\neq i}^{\kappa_{n}}e^{-\gamma (1-p_{k})}+\sum_{k=\kappa_{n}+1,k\neq n}^{n}e^{-\gamma (1+p_{n-k+\kappa_{n}+1})}}. \end{array}$$

Therefore, according Formula (A14), if it wants to show $\frac{q_{ij}}{q_{ij^{\prime }}}\leq e^{\gamma (1-p_{i+1})}$, then it needs to show $1+e^{-\gamma (1-p_{i})}\leq e^{\gamma (p_{i}-p_{i+1})}+e^{\gamma (p_{i}-p_{i+1})}e^{-\gamma (1+p_{\kappa_{n}+1})}$, i.e.,

(A15) $$1+e^{-\gamma (1-p_{i})}-e^{\gamma (p_{i}-p_{i+1})}-e^{\gamma (p_{i}-p_{i+1})}e^{-\gamma (1+p_{\kappa_{n}+1})}\leq 0$$

always stands up. Let $f\left(x\right)=1+e^{-\gamma (1-x)}-e^{\gamma (x-p_{i+1})}-e^{\gamma (x-p_{i+1})}e^{-\gamma (1+p_{\kappa_{n}+1})}$, where $x\in [0,p_{\kappa_{n}}],\gamma >0$ and $0\leq p_{i+1}\leq x$. If it takes the derivative of f(x) with respect to *x*, it gets $f{\left(x\right)}^{\prime }=\gamma e^{-\gamma (1-x)}-\gamma e^{\gamma (x-p_{i+1})}-\gamma e^{-\gamma (1+p_{\kappa_{n}+1})}e^{\gamma (x-p_{i+1})}\leq 0$, i.e., f(x) decreases monotonically with *x*. It implies that f(x)≤f(0)=0 always stands up. Therefore, the inequation $1+e^{-\gamma (1-p_{i})}-e^{\gamma (p_{i}-p_{i+1})}-e^{\gamma (p_{i}-p_{i+1})}e^{-\gamma (1+p_{\kappa_{n}+1})}\leq 0$ always stands up for $p_{i}\in \left[0,p_{k_{n}}\right]$. From the above discussion, it can be seen that

(A16) $$\begin{array}{c} \frac{q_{ij}}{q_{ij^{\prime }}}\leq \frac{q_{ii}}{q_{in}}\leq e^{\gamma (1-p_{i+1})}. \end{array}$$

Similarly, it has

(A17) $$\begin{array}{cc} \frac{q_{ij}}{q_{ij^{\prime }}} & \geq \frac{q_{ij}}{q_{ii}}\geq \frac{q_{in}}{q_{ii}}\geq e^{-\gamma (1-p_{i+1})}. \end{array}$$

(ii) For $n>i>\kappa_{n}$, $\kappa_{n}\leq n-2$ and $j,j^{\prime }\in \left[1,n\right]$, it has

(A18) $$\begin{array}{cc} \; & \frac{q_{ij}}{q_{ij^{\prime }}}\leq \frac{q_{ii}}{q_{in}}\\ \; & =\frac{\frac{1}{\Omega_{i}}}{\frac{e^{-\gamma (1+p_{n-i+\kappa_{n}+1})}}{\Omega_{n}}}\\ \; & =e^{\gamma (1+p_{n-i+\kappa_{n}+1})}\frac{\Omega_{n}}{\Omega_{i}}\\ \; & =e^{\gamma (1+p_{n-i+\kappa_{n}+1})}\frac{1+\sum_{k=1}^{\kappa_{n}}e^{-\gamma (1-p_{k})}+\sum_{k=\kappa_{n}+1,k\neq n}^{n}e^{-\gamma (1+p_{n-k+\kappa_{n}+1})}}{1+\sum_{k=1}^{\kappa_{n}}e^{-\gamma (1-p_{k})}+\sum_{k=\kappa_{n}+1,k\neq i}^{n}e^{-\gamma (1+p_{n-k+\kappa_{n}+1})}}\\ \; & \leq e^{\gamma (1+p_{n-i+\kappa_{n}})}\left(1+e^{-\gamma (1+p_{n-i+\kappa_{n}+1})}+\sum_{k=1}^{\kappa_{n}}e^{-\gamma (1-p_{k})}+\sum_{k=\kappa_{n}+1,k\neq i,k\neq n}^{n}e^{-\gamma (1+p_{n-k+\kappa_{n}+1})}\right)\times \\ \; & \frac{1}{e^{\gamma (p_{n-i+\kappa_{n}}-p_{n-i+\kappa_{n}+1})}\left(1+e^{-\gamma (1+p_{\kappa_{n}+1})}\right)+\sum_{k=1}^{\kappa_{n}}e^{-\gamma (1-p_{k})}+\sum_{k=\kappa_{n}+1,k\neq i,k\neq n}^{n}e^{-\gamma (1+p_{n-k+\kappa_{n}+1})}}. \end{array}$$

Hence, if we want to show $\frac{q_{ij}}{q_{ij^{\prime }}}\leq e^{\gamma (1+p_{n-i+\kappa_{n}})}$ according the Formula (A18), then it needs to show that $1+e^{-\gamma (1+p_{n-i+\kappa_{n}+1})}$$\leq e^{\gamma (p_{n-i+\kappa_{n}}-p_{n-i+\kappa_{n}+1})}\left(1+e^{-\gamma (1+p_{\kappa_{n}+1})}\right)$, i.e.,

(A19) $$1+e^{-\gamma (1+p_{n-i+\kappa_{n}+1})}-e^{\gamma (p_{n-i+\kappa_{n}}-p_{n-i+\kappa_{n}+1})}\left(1+e^{-\gamma (1+p_{\kappa_{n}+1})}\right)\leq 0$$

always stand up. Let $f\left(x\right)=1+e^{-\gamma (1+x)}-e^{\gamma (p_{n-i+\kappa_{n}}-x)}\left(1+e^{-\gamma (1+p_{\kappa_{n}+1})}\right)$, where $x\in \left[p_{n},p_{k_{n}+1}\right]\left(i\in \left[k_{n}+1,n\right]\right),\gamma >0$ and $p_{n-i+\kappa_{n}}\geq x$. If we take the derivative of f(x) with respect to *x*, we get $f{\left(x\right)}^{\prime }=-\gamma e^{-\gamma (1+x)}+\gamma e^{\gamma (p_{n-i+\kappa_{n}}-x)}\left(1+e^{-\gamma (1+p_{\kappa_{n}+1})}\right)\geq 0$, that is, f(x) increases monotonically with *x*. Then $f\left(x\right)\leq f\left(p_{k_{n}+1}\right)=1+e^{-\gamma (1+p_{k_{n}+1})}-e^{\gamma (p_{n-i+\kappa_{n}}-p_{k_{n}+1})}\left(1+e^{-\gamma (1+p_{\kappa_{n}+1})}\right)\leq 0$ always stand up. Therefore, the inequation $1+e^{-\gamma (1+p_{n-i+\kappa_{n}+1})}-e^{\gamma (p_{n-i+\kappa_{n}}-p_{n-i+\kappa_{n}+1})}\left(1+e^{-\gamma (1+p_{\kappa_{n}})}\right)\leq 0$ always stand up for $p_{n-i+\kappa_{n}+1}$$\in \left[p_{n},p_{k_{n}+1}\right)\left(i\in \left[k_{n}+1,n\right)\right)$. Similarly, it has

(A20) $$\begin{array}{cc} \frac{q_{ij}}{q_{ij^{\prime }}} & \geq \frac{q_{ij}}{q_{ii}}\geq \frac{q_{in}}{q_{ii}}\geq e^{-\gamma (1+p_{n-i+\kappa_{n}})}. \end{array}$$

(iii) For i=n and $j,j^{\prime }\in \left[1,n\right]$, it has

(A21) $$\begin{array}{cc} \frac{q_{nj}}{q_{nj^{\prime }}} & \leq \frac{q_{nn}}{q_{n(n-1)}}\\ \; & =\frac{\frac{1}{\Omega_{n}}}{\frac{e^{-\gamma (1+p_{\kappa_{n}+1})}}{\Omega_{(}n-1)}}\\ \; & =e^{\gamma (1+p_{\kappa_{n}+1})}\frac{\Omega_{n-1}}{\Omega_{n}}\\ \; & =e^{\gamma (1+p_{\kappa_{n}+1})}\frac{1+e^{-\gamma (1+p_{\kappa_{n}+1})}+\sum_{k=1}^{\kappa_{n}}e^{-\gamma (1-p_{k})}+\sum_{k=\kappa_{n}+1,k\neq n-1,k\neq n}^{n}e^{-\gamma (1+p_{n-k+\kappa_{n}+1})}}{1+e^{-\gamma (1+p_{\kappa_{n}+2})}+\sum_{k=1}^{\kappa_{n}}e^{-\gamma (1-p_{k})}+\sum_{k=\kappa_{n}+1,k\neq n-1,k\neq n}^{n}e^{-\gamma (1+p_{n-k+\kappa_{n}+1})}}\\ \; & \leq e^{\gamma (1+p_{\kappa_{n}+1})}. \end{array}$$

Similarly, it has

(A22) $$\begin{array}{cc} \frac{q_{nj}}{q_{nj^{\prime }}} & \geq \frac{q_{nj}}{q_{nn}}\geq \frac{q_{n(n-1)}}{q_{nn}}\geq e^{-\gamma (1+p_{\kappa_{n}+1})}. \end{array}$$

From the above discussion of (i)∼(iii), it can be seen that the part (1) of Theorem A2 holds. □
